# The iron-sulfur accelerator YgfZ modulates genome-wide IHF-binding dynamics to regulate replication initiation in *Escherichia coli*

**DOI:** 10.3389/fmicb.2026.1781011

**Published:** 2026-03-17

**Authors:** Kazutoshi Kasho, Rion Satomura, Mizuki Yoshida, Ryuki Murofushi, Ikuha Kitamura, Sho Nakagawa, Wataru Nakagaki, Tsutomu Katayama

**Affiliations:** Department of Molecular Biology, Graduate School of Pharmaceutical Sciences, Kyushu University, Fukuoka, Japan

**Keywords:** datA, DnaA, *Escherichia coli*, IHF, iron-sulfur cluster, replication initiation, YgfZ

## Abstract

In *Escherichia coli*, chromosome replication is regulated through ATP/ADP state of the DnaA initiator. The DDAH system inactivates DnaA in the post-initiation stage by promoting ATP hydrolysis through timely binding of the DNA-bending protein IHF to the *datA* locus, while the *DARS2* locus reactivates DnaA in the pre-initiation stage via binding of IHF and another nucleoid protein Fis. The iron-sulfur cluster [(Fe-S)] assembly factor YgfZ is known to sustain replication initiation, central carbon metabolism, redox state and modification of tRNA A37 residues by MiaB, but the link between initiation and the others remains unclear. This study shows that YgfZ regulates initiation primarily by downregulating the DDAH system by repressing *datA*-IHF binding in a manner independent of MiaB. Also, the [Fe-S]-binding protein MnmA moderately downregulates *datA*-IHF binding. Furthermore, YgfZ globally downregulates basal IHF binding across the genome, while preserving IHF's timely binding at key loci including *oriC* and *datA* during the cell cycle, highlighting a novel strategy: YgfZ modulates both the cellular metabolic states and global genome dynamics to control replication initiation under various growth conditions.

## Introduction

**Figure 1:**
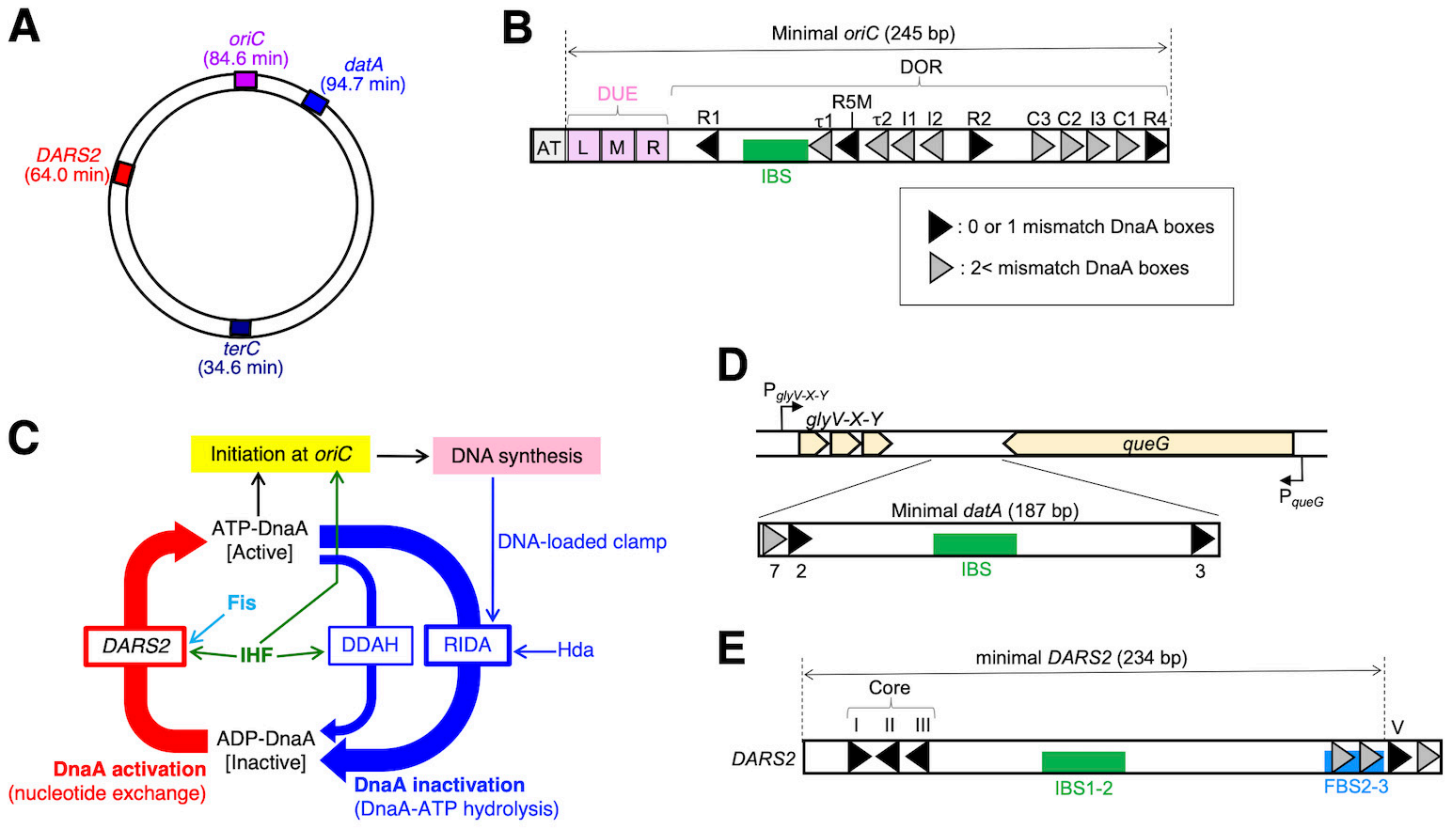
Overview of regulatory mechanisms of E. coli replication initiation. **(A)** Schematic presentation of the genomic DNA elements *oriC, datA, DARS2*, and replication termination site *terC* on the 4.6 Mb circular *E. coli* chromosome. **(B)** Structure of *oriC*. The open bar indicates oriC (250 bp) including 9-mer DnaA boxes (black or gray triangles), IBS (green bar), the DUE including the 13-mer elements (pink squares), and DnaA oligomerization region (DOR). **(C)** Schematic presentation of the regulatory cycle of DnaA. ATP-DnaA initiates genomic DNA replication from *oriC* with the aid of IHF. ATP-DnaA is converted to ADP-DnaA (RIDA and DDAH systems). RIDA requires a complex between Hda protein and the DNA-loaded form of the clamp subunit of DNA polymerase III holoenzyme, and DDAH requires the *datA-IHF* complex. *DARS2* loci stimulate nucleotide exchange of ADP-DnaA to produce ATP-DnaA. *DARS2*-dependent ATP-DnaA production requires IHF and Fis. **(D)** A schematic presentation of the genomic datA locus. Genes or ORFs (*glyV-X-Y* and *queG*) are indicated by gray arrows. Bent arrows indicate transcriptional promoters (*PglyV-X-Y* and *PqueG*). The minimal *datA* (187 bp) consists of DnaA boxes 2, 3, and 7 and a single IBS, which is located between *glyV-X-Y* and *queG*. The *datA* DnaA box 4 stimulates DDAH *in vitro*. **(E)** Structures of DARS2. The open bar indicates DARS2 (256 bp). Black or gray arrowheads represent DnaA boxes. DARS2 carries the oppositely-oriented DnaA boxes I, II, and III, termed the core, which is essential for DnaA-ADP dissociation. Green and sky blue bars represent IBS1-2 and FBS2-3, respectively, both of which are also essential for *DARS2* activation.

The accurate and timely replication of chromosomes is universally conserved and indispensable for cell proliferation. A hallmark of this essential process is its precise regulation, ensuring that DNA synthesis initiates precisely once per cell cycle. In *Escherichia coli*, this timely initiation is driven by the binding of initiation-active ATP form of DnaA and IHF, a nucleoid associated protein (NAP), to the replication origin *oriC* (Costa et al., 2013; Grimwade and Leonard, 2021; Kasho et al., 2023) (Figures 1A, B). DnaA consists of four distinct domains. The N-terminal domain I is involved in protein–protein interactions (Abe et al., 2007; Keyamura et al., 2009; Sutton et al., 1998; Zawilak-Pawlik et al., 2017). Domain II is a flexible linker (Nozaki and Ogawa, 2008). Domain III contains AAA+ (ATPase associated with various cellular activities) motifs involved in ATP/ADP binding, ATP hydrolysis, ss (single-stranded) *oriC* DNA binding and assembly of DnaA molecules (Duderstadt et al., 2011; Kawakami et al., 2005; Noguchi et al., 2015; Saxena et al., 2015). Domain IV binds specifically to the 9-mer DnaA box, TTAWnCACA (Fujikawa et al., 2003). IHF, a heterodimer of IHFα and IHFβ subunits (encoded by *ihfA* and *ihfB*, respectively) specifically binds to its 13-mer consensus sequence (TAAnnnnTTGATW, where W is A or T, and n is any nucleotide) and sharply bends DNA (Aeling et al., 2006; Dillon and Dorman, 2010). *oriC* comprises the duplex unwinding element (DUE), a DnaA oligomerization region (DOR) including 9-mer DnaA binding consensus sequences (DnaA boxes: TTATnCACA), its derivatives with a single or few mismatch(es) and a single IHF-binding site (IBS) (Duderstadt et al., 2011; Sakiyama et al., 2022; Saxena et al., 2015) (Figure 1B). Ordered assembly of ATP-DnaA molecules and IHF on *oriC*, resulting in an initiation complex that unwinds the DUE to load DnaB helicase. The initiation activity of DnaA is tightly regulated by its bound nucleotide (ATP or ADP), with the cellular level of ATP-DnaA peaking at approximately 80% at the initiation time of the replication cycle (Kurokawa et al., 1999). However, despite this established framework, a significant gap persists in our understanding of how cellular ATP-DnaA levels are precisely tuned in response to the myriads of physiological conditions and varying growth rates experienced by cells. Bridging this knowledge gap is fundamental to deciphering the adaptability and robustness of bacterial cell cycle control.

Specifically after initiation, ATP-DnaA is converted into initiation-inactive ADP-DnaA by two distinct systems, RIDA (regulatory inactivation of DnaA) and DDAH (*datA*-dependent DnaA-ATP hydrolysis) (Kasho and Katayama, 2013; Katayama et al., 1998; Kim et al., 2017) (Figure 1C). In RIDA, the complex of the AAA+ family protein Hda and the DNA-loaded form of the clamp (β subunit) of the DNA polymerase III holoenzyme interacts catalytically with ATP-DnaA molecules, promoting ATP hydrolysis to produce ADP-DnaA (Katayama et al., 1998; Kim et al., 2017). DDAH is activated by the formation of a complex between the chromosomal *datA* locus and IHF (Kasho and Katayama, 2013) (Figures 1C, D). The minimal *datA* sequence essential for DDAH activity contains three DnaA boxes and a single IBS (Kasho et al., 2017). The formation of the *datA*-DnaA-IHF tripartite complex induces specific inter-DnaA interactions, stimulating the DnaA-ATP hydrolysis (Kasho et al., 2017; Kasho and Katayama, 2013). IHF binding to *datA* is remarkably cell cycle-dependent, occurring predominantly at the post-initiation stage and dissociating prior to initiation (Kasho and Katayama, 2013). Our recent study revealed that transcription initiated from the upstream tRNA-Gly (*glyV-X-Y*) operon reads through *datA* and this is required for dissociation of *datA*-IHF complex (Kasho et al., 2024) (Figure 1D). However, the precise mechanism driving timely *datA*-IHF binding/dissociation remains to be investigated.

To initiate a new round of replication in a timely manner, ADP-DnaA must be efficiently converted to ATP-DnaA at the pre-initiation stage by the chromosomal *DARS2* (DnaA-reactivating sequence 2) locus (Fujimitsu et al., 2009; Kasho et al., 2014) (Figures 1C, E). *DARS2* carries core DnaA boxes and specific binding sites for its activators: IHF and another NAP Fis, corresponding to IBS1-2 and FBS2-3, respectively (Kasho et al., 2014, 2025). Fis is expressed in an exponential growth phase-specific manner and specifically binds to its consensus sequence (GnnYAnnnnTRnnC, where Y is T or C, and R is A or G), inducing shallow DNA curvature (Ball et al., 1992; Stella et al., 2010). ADP-DnaA molecules bound to the core DnaA boxes and those bound to DnaA boxes I and II interact with each other through its domain III in a head-to-head manner (Figure 1E), driving the crucial ADP-to-ATP exchange of these molecules to regenerate ATP-DnaA (Fujimitsu et al., 2009; Sugiyama et al., 2019). In addition, ^29, 34^the activation of *DARS2* critically depends on the simultaneous binding of IHF and Fis to *DARS2* IBS1-2 and FBS2-3 at the pre-initiation stage, which stimulates ATP-DnaA production at the core region, ensuring precise initiation timing (Kasho et al., 2014). Next, at the initiation stage, ATP-DnaA molecules are accumulated so as to form oligomers in the region spanning the *DARS2* DnaA box V to its flanking sites which overlap with the FBS2-3 (Figures 1C, E). This causes dissociation of Fis by binding competition, exerting negative feedback for *DARS2* function for prohibiting excess accumulation of ATP-DnaA (Miyoshi et al., 2021). As such, regulation for Fis is well-defined. However, in contrast to this, regulation of IHF at *datA* or *DARS2* remains an outstanding question, presenting a significant gap in our understanding of the cell cycle control.

**Figure 2:**
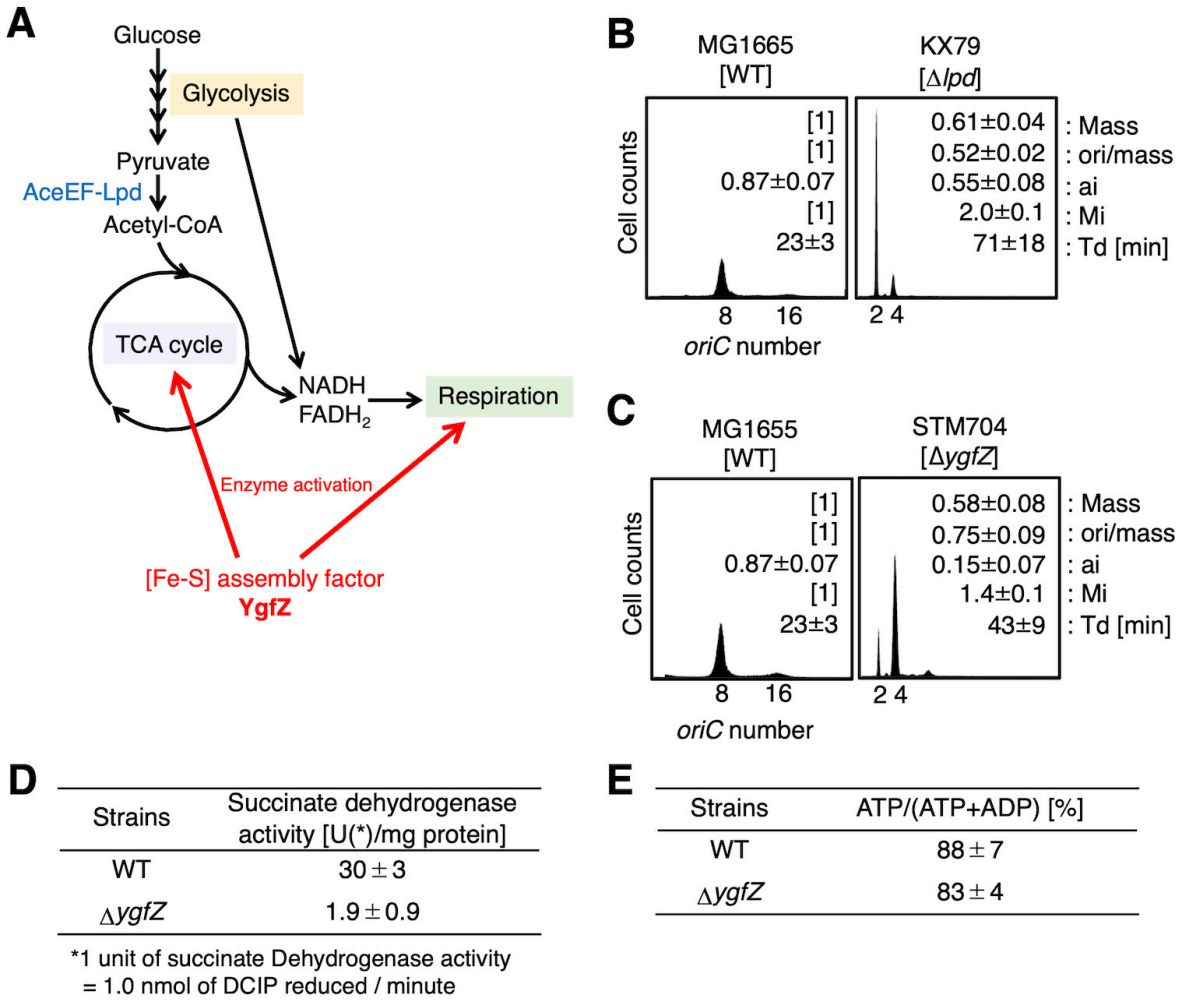
Involvement of carbon metabolism enzymes and [Fe-S] maintenance factor YgfZ in the regulation of the timing of replication initiation. **(A)** Schematic presentation of the glucose metabolism pathways in *E. coli*. The glycolysis enzymes convert glucose into pyruvate, which is further converted to acetyl-CoA by the pyruvate dehydrogenase complex (AceEF-Lpd) and enters the TCA cycle. The products of the TCA cycle NADH and FADH2 are utilized by aerobic respiration for effective ATP production. In the described glucose metabolism system, the [Fe-S] maintenance factor *YgfZ* stimulates several enzymes in TCA cycle and respiration pathways. **(B)** MG1655 [WT] or KX79 [Δ*lpd*] cells were cultivated at 37 °C in LB medium and analyzed by flow cytometry. The doubling times (Td) [min], relative ratios of cell mass, ori/mass, initiation age (ai), and initiation mass (Mi) (set MG1655 cells [1] as a standard) are shown in the histogram. Errors represent the standard deviations calculated from three independent experiments. **(C)** MG1655 [WT] and STM704 [Δ*ygfZ*] cells were analyzed. Errors represent the standard deviations calculated from seven (MG1655) or fifteen (STM704) independent experiments. **(D)** Effect of deletion of *ygfZ* on succinate dehydrogenase activity. MG1655 [WT] and STM704 [Δ*ygfZ*] cells were cultivated at 37 °C in LB medium until A660~0.5, and the membrane vesicle fractions were prepared according to Waller et al. (2010) and analyzed using the Succinate Dehydrogenase Assay Kit (Abcam). Two independent experiments were performed, and the mean values and errors are indicated. **(E)** Effect of deletion of *ygfZ* on the cellular ATP level. MG1655 [WT] and STM704 [Δ*ygfZ*] cells were cultivated at 37 °C in LB medium until A660~0.1, and analyzed the ATP level using ADP/ATP Ratio Assay Kit-Luminescence (DOJINDO).

Central carbon metabolism (CCM) is indispensable for the ATP production needed for cell growth and is important for environmental adaptation (Figure 2A). In *E. coli*, CCM is an adaptable network of various pathways including glycolysis, TCA cycle, respiration and fermentation, and is dynamically adjusted in response to nutrient availability, ensuring robust cell proliferation across diverse growth conditions. [Fe-S]-containing enzymes are important to these cellular functions. YgfZ is a bacterial homolog of yeast/human mitochondrial IBA57 protein which interacts with the core [Fe-S] assembly protein ISCA and stimulates the assembly of [Fe-S] cluster Imlay, (2006); Lénon et al., (2022); Mühlenhoff et al., (2022); Waller et al., (2012a), a constituent of certain TCA cycle enzymes (Waller et al., 2010). Consistently, deletion of *ygfZ* reduces the activities of the [Fe-S]-dependent TCA cycle enzymes, succinate dehydrogenase and fumarase (Waller et al., 2010), and growth defects of *ygfZ*-deleted (Δ*ygfZ*) cells are complemented with the eukaryotic IBA57 homologs (Waller et al., 2010) (Figure 2A). Intriguingly, our previous work identified YgfZ as a suppressor of temperature-sensitive mutants of *hda*, a constituent of RIDA system, with Δ*ygfZ* cells exhibiting reduced levels of ATP-DnaA(Ote et al., 2006), suggesting that YgfZ contributes to the regulation of DnaA activity possibly via *DARS2* or DDAH. Consistently, iron chelation also represses excess initiations caused by *hda* deletion or *DARS2* oversupply (Charbon et al., 2018). However, the precise mechanistic relationship between YgfZ-dependent [Fe-S] assembly and DnaA regulation remains obscure.

The situation is further complicated by the fact that YgfZ is important for the activity of [Fe-S] cluster enzymes involved in the regulation of tRNA A37 residue or ribosomal protein S12 modification (Bak and Weerapana, 2023; Lund et al., 2023; Waller et al., 2010). Briefly, deletion of *ygfZ* diminishes MiaB-dependent tRNA ms^2^i^6^A37 (2-methylthio *N*^6^- isopentenyladenosine) modification as well as RimO-dependent S12 thiomethylation. Interestingly, deletion of *mnmE*, another tRNA modification enzyme involved in mcm^5^s^2^U34 (5-methoxycarbonylmethyl-2-thiouridine) modification, partially restores ms^2^i^6^A37 modification and suppresses the growth defect observed in Δ*ygfZ* cells (Lund et al., 2023; Ote et al., 2006; Waller et al., 2012b). In addition, the *E. coli* MnmA promotes sulfation of the wobble uridine (mcm^5^s^2^U34) of tRNA in both a [Fe-S]-dependent and independent manners (Leimkühler, 2025; Zhou et al., 2021). Like deletion of *ygfZ*, deletion of *mnmA* impacts the cellular redox state and TCA cycle activity (Nakayashiki et al., 2013). Collectively, these observations suggest an unexplored link between replication initiation and the cellular redox state, CCM or tRNA modification via [Fe-S] cluster proteins.

In this study, we revealed that YgfZ stimulates replication initiation, downregulating the DDAH system through repressing *datA*-IHF binding. Mutant analysis indicated that the regulation is independent of tRNA modification. Notably, using both local and genome-wide analysis of IHF binding, we found that, in Δ*ygfZ* cells, genomic IHF binding was broadly increased regardless of the location at gene body or intergenic region, and especially the binding was highly enhanced at the regions where IHF binding is inherently weak (e.g., *rrn* operons, tRNA gene-proximal regions, among others). Taken together, these results suggest that YgfZ-dependent [Fe-S] assembly is important in genome-wide regulation for IHF binding, which modulates specific genome functions such as replication initiation under various growth conditions. MnmA also moderately downregulates *datA*-IHF binding, supporting importance of [Fe-S] cluster proteins for integrating metabolic state with genome dynamics.

## Materials and methods

### DNA, *E. coli* strains, and cultures

All *E. coli* strains used in this study are listed in Table 1. Δ*lpd*::*frt-kan* (JW0112-KC), Δ*ygfZ*::*frt-kan* (JW2866-KC), Δ*mnmA*::*frt-kan* (JW1119-KC), Δ*miaB*::*frt-kan* (JW0658-KC), Δ*mnmC*::*frt-kan* (JW5380-KC), Δ*mnmE*::*frt-kan* (JW3684-KC), and Δ*mnmG(gidA)*::*frt-kan* (JW3719-KC) mutations were derived from the Keio collection and introduced into MG1655 cells using P1 transduction, yielding KX79, STM704, STM705, STM706, STM701, STM702, and STM703, respectively. The *kan* gene was removed from STM705 by pCP20, yielding KSZ3. Δ*ygfZ*::*frt-kan* was introduced into KSZ3, SA182, MIT78, and KYA018 cells by P1 transduction, yielding MYK2 [Δ*ygfZ* Δ*mnmA*], STM708 [Δ*ygfZ* Δ*datA*], STM707 [Δ*ygfZ* Δ*DARS2*], and STM715 [*dnaC2* Δ*ygfZ*], respectively. Δ*DARS2*::*spec* was introduced into STM708 cells by P1 transduction, yielding IKH17 [Δ*ygfZ* Δ*datA*Δ*DARS2*]. Δ*ygfZ*::*frt-kan* or Δ*mnmA*::*frt-kan* was introduced into KX200 [*ihfA-cHis*_12_] cells by P1 transduction, yielding KX283 [*ihfA-cHis*_12_Δ*ygfZ*] and KX293 [*ihfA-cHis*_12_Δ*mnmA*], respectively.

**Table 1:** List of E. coli strains used in this study.

**Strain**	**Genotype**	**Source**
MG1655	WT	Laboratory stock
DH5α	s*upE44*Δ*lacU169(ϕ80lacZ*Δ*M15)*	Laboratory stock
	*hsdR17 recA1 endA1 gyrA96 thi-1 relA1*	
BW25112	*rrnB DElacZ4787 HsdR514 DE(araBAD)567 DE(rhaBAD)568 rph-1*	Keio Collection (NBRP)
JW0112-KC	BW25112 Δ*lpd*::*frt-kan*	Keio Collection (NBRP)
JW2866-KC	BW25112 Δ*ygfZ*::*frt-kan*	Keio Collection (NBRP)
JW1119-KC	BW25112 Δ*mnmA*(*trmU*)::*frt-kan*	Keio Collection (NBRP)
JW0658-KC	BW25112 Δ*miaB*::*frt-kan*	Keio Collection (NBRP)
JW5380-KC	BW25112 Δ*mnmC*::*frt-kan*	Keio Collection (NBRP)
JW3684-KC	BW25112 Δ*mnmE*(*trmE*)::*frt-kan*	Keio Collection (NBRP)
JW3719-KC	BW25112 Δ*mnmG*(*gidA*)::*frt-kan*	Keio Collection (NBRP)
KX79	MG1655 Δ*lpd*::*frt-kan*	This work
STM704	MG1655 Δ*ygfZ*::*frt-kan*	This work
MYK1	MG1655 Δ*mnmA*::*frt-kan*	This work
STM706	MG1655 Δ*miaB*::*frt-kan*	This work
STM701	MG1655 Δ*mnmC*::*frt-kan*	This work
STM702	MG1655 Δ*mnmE*::*frt-kan*	This work
STM703	MG1655 Δ*mnmG*::*frt-kan*	This work
KSZ3	MG1655 Δ*ygfZ*::*frt*	This work
MYK2	KSZ3 Δ*mnmA*::*frt-kan*	This work
SA182	MG1655 Δ*datA*::*cat*	Kasho et al., 2017
MIT78	MG1655 Δ*DARS2*::*cat*	Fujimitsu et al., 2009
IKH16	SA182 Δ*DARS2*::*spec*	This work
STM708	STM704 Δ*datA*::*cat*	This work
STM707	STM704 Δ*DARS2*::*cat*	This work
IKH17	STM708 Δ*DARS2*::*spec*	This work
KYA018	MG1655 *dnaC2 zjj-18::cat*	Kasho and Katayama, 2013
STM715	KYA018 Δ*ygfZ*::*frt-kan*	This work
KX200	MG1655 *ihfA-cHis12*	This work
KX283	KX200 Δ*ygfZ*::*frt-kan*	This work
KX293	KX200 Δ*mnmA*::*frt-kan*	This work

### Flow cytometry

Flow cytometry experiments were performed according to a previously described method (Kasho et al., 2024, 2025). Briefly, cells were cultivated at 37 °C in LB medium or supplemented M9 medium in the presence or absence of 50 μg/mL ampicillin until an A_660_ (absorbance at 660 nm) of 0.1-0.2 was reached, followed by further incubation at 37 °C for 4 h in the presence of 300 μg/mL rifampicin and 10 μg/mL cephalexin for run-out replication. The resultant cells were fixed with 70% ethanol, stained with SYTOX Green (Life Technologies), and analyzed the average values of cell volume (mass) and *oriC* number using FACS Calibur or FACS Lyric (BD Biosciences). The initiation parameters (initiation age ai, and initiation mass Mi) was calculated according the previously described equations (Wold et al., 1994).

### Immunoblotting

Immunoblotting experiments were performed according to a previously described method (Kasho et al., 2014). Briefly, proteins in OD_660_ × vol [μL] = 100 of each cell-culture sample were separated by SDS-20% PAGE and analyzed by western blotting using anti-IHF antiserum. Band intensities were quantified, and background intensity was subtracted.

### Chromatin Immunoprecipitation and chromatin affinity purification with quantitative PCR (ChIP-qPCR and ChAP-qPCR)

ChIP, ChAP, and qPCR experiments were performed according to a previously described method (primers are listed in Table 2) (Kasho et al., 2014; Kasho and Katayama, 2013).

**Table 2:** List of oligonucleotides used in this study.

**Names**	**Sequences**
ORI_1	CTGTGAATGATCGGTGATC
KWoriCRev	GTGGATAACTCTGTCAGGAAGCTTG
IHF-D2F	GTCACACCTCTCATTTACGGG
IHF-D2B	CCAGTTTTTAGTGGTTCAGTGC
RT-datAIBS_L	CAGAGTTATCCACAGCCTCAGG
RT-datAIBS_R	CAAGTGATCGACTCGACAAAAC
RTYLCC-L	GGCGTGGTAAAGGGTATCG
RTYLCC-R	TCTGCGGGGTGATGGTAAAG
ilvG-U	TCCTCGGTTATGTTTTTAAGGTC
ilvG-L	TGCACTTGGACGAGGAAAG
rhlB-U	TACGTCACGACCCGCCAG
rhlB-L	CATCCGAAGGTTGTAGAAGC
glnH-U	AATGGTGCATCTTCAGGGTATTG
glnH-L	CACATATATGAAAAAATCGTGCCAG
osmY-U	ATCACAATTTTGAAACCGCTC
osmY-L	CTGTCAATTTCCCTTCCTTATTAGC

### ChAP with high-throughput sequencing (ChAP-seq)

ChAP experiments were performed with minor modifications (Kasho et al., 2014; Kasho and Katayama, 2013). Briefly, KX200 [*ihfA-cHis*_12_] or KX283 [*ihfA-cHis*_12_ Δ*ygfZ*] cells were grown in LB medium (200 mL) at 37 °C until an A_660_ of 0.1-0.2 was reached. They were then crosslinked, collected by centrifugation, and washed with 1 mL of ice-cold TBS [50 mM Tris-HCl (pH7.5) and 500 mM NaCl]. The cells were resuspended in 1 mL of binding buffer [50 mM Tris-HCl (pH 7.5), 500 mM NaCl, 1% (vol/vol) Triton X-100, 5 mM imidazole, and 100 μM PMSF] and sonicated for a total ON time of 4 min (ON 10 sec, OFF 20 sec) each. The cell debris was then removed by centrifugation, and a portion (950 μL) of the resulting supernatant was mixed with 100 μL of Dynabeads His-tag Isolation & Pulldown (Life Technologies), followed by incubation at 4 °C for 2 h with gentle rotation. Beads and bound materials were washed four times with wash buffer [50 mM Tris-HCl (pH 7.5), 500 mM NaCl, 1% (vol/vol) Triton X-100, and 5 mM imidazole], resuspended in elution buffer [50 mM Tris-HCl (pH 7.5), 500 mM imidazole, 1% SDS, 10 mM EDTA, and 10 mM dithiothreitol], and incubated at 65 °C for 12 h to allow de-crosslinking. DNA in the samples before (Input) and after (ChAP) pull down was purified using a MagExtractor™ –PCR & Gel Clean up– (TOYOBO). To check the quality of the samples, the levels of *oriC, datA, DARS2*, and *ylcC* were quantified by real-time qPCR. DNA library preparation and next-generation sequencing were performed by Azenta Life Sciences (Massachusetts, US) on the Illumina Novaseq platform, in a 2 × 150 bp paired-end configuration, with at least 20 Mb reads per sample. The qualities of obtained data were checked by FastQC, and alignment and peak calling were performed on Galaxy (bowtie and macs, respectively) (Galaxy Community, 2024). The peaks were then visualized using the IGV (Integrative Genome Viewer) genome browser. Motif analysis of the obtained IHF binding sites in each sample was performed using MEME-ChIP.

## Results

### YgfZ controls the timing of replication initiation in response to glucose metabolism

To unravel the interplay between CCM, [Fe-S] metabolism, and the regulation of replication initiation, we first performed flow cytometry to analyze the initiation profiles (ori/mass and initiation mass) of Lpd (lipoamide dehydrogenase), a core CCM enzyme (Figures 2A, B). The ori/mass is the number of *oriC* per cell volume (mass), and the initiation mass (Mi) is the cell volume per *oriC* at the initiation time (Wold et al., 1994). Lpd is a crucial component of the pyruvate dehydrogenase complex (AceEF-Lpd), serving as a link connecting glycolysis to the TCA cycle (Li et al., 2006; Quail et al., 1994), and consistent with previous report (Li et al., 2006), Δ*lpd* cells exhibit significantly reduced growth rates (doubling time, Td = 71 ± 18 min) compared with wild-type (WT) cells (Td = 23 ± 3 min). Consistently, flow cytometry analysis revealed that Δ*lpd* cells cultivated in LB medium displayed hallmarks of impaired initiation (ori/mass = 0.52 ± 0.02, Mi = 2.0 ± 0.1, set WT ori/mass and Mi as a standard [1]), further suggesting a link between CCM states and replication initiation.

**Figure 3:**
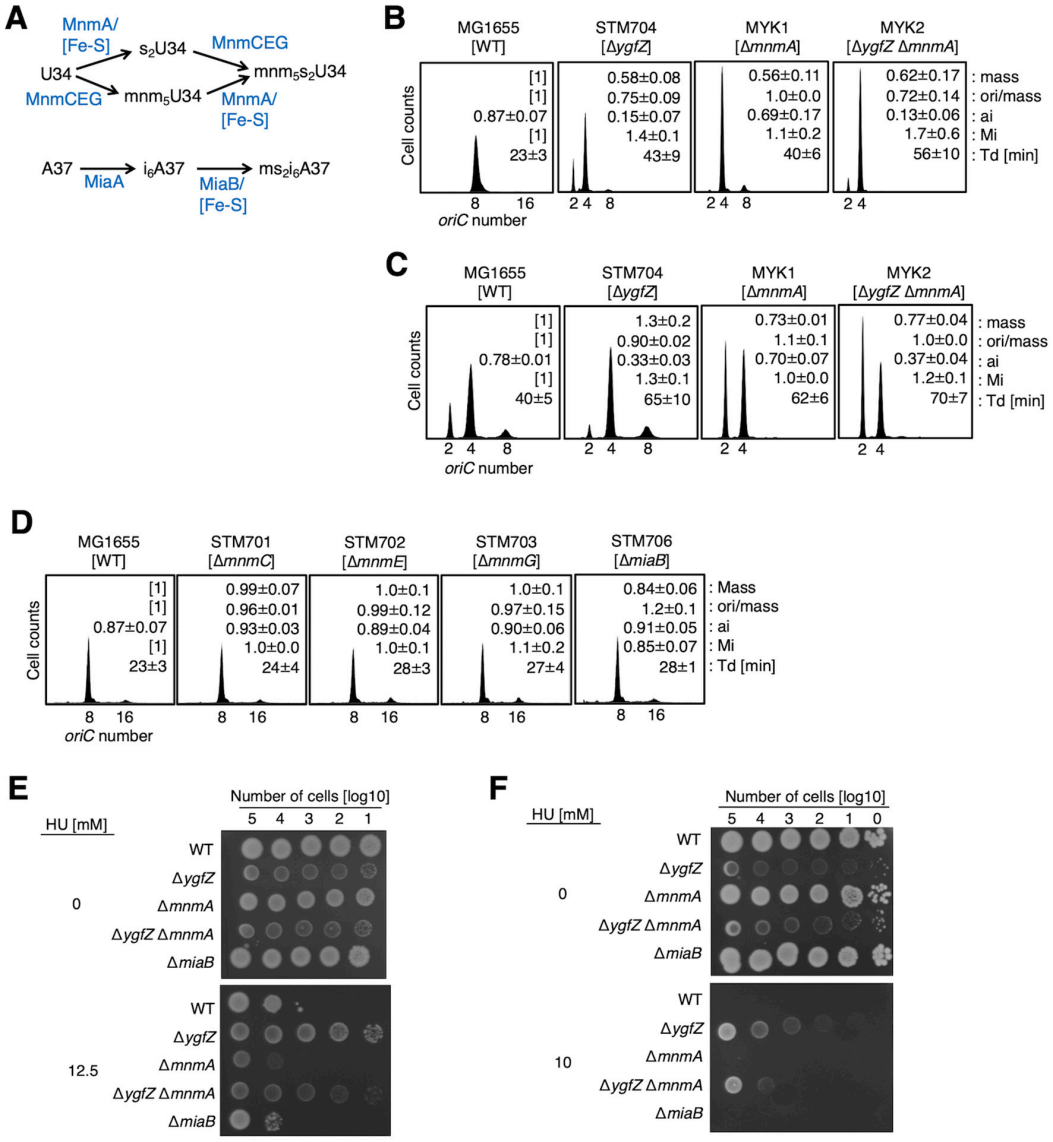
Analysis of tRNA modification in the YgfZ-dependent stimulation of replication initiation. **(A)** Schematic presentation of the [Fe-S]-dependent tRNA modification pathways in *E. coli*. MnmA incorporates sulfur and MnmCEG pathway incorporates mnm^5^ modification at U34 to form mnm^5^s^2^U. MnmC is a bifunctional enzyme that catalyzes the de-modification of cmnm^5^(s^2^)U34 to nm^5^(s^2^)U34 in a manner dependent on FAD, and the following transfer of a methyl group from S-adenosyl-L-methionine to nm^5^(s^2^)U34 to form mnm^5^(s^2^)U34. The functional complex of MnmE and MnmG synthesizes cmnm^5^(s^2^))U and nm^5^(s^2^)U using glycine and ammonium, respectively. MiaB catalyzes the methyl-thiolation of i^6^A to form ms^2^i^6^A at position 37 in tRNA. **(B)** MG1655 [WT], KSZ3 [Δ*ygfZ*], MYK1 [ΔmnmA], and MYK2 [Δ*ygfZ* Δ*mnmA*] cells were cultivated at 37 °C in LB medium **(B)** or in M9 medium supplemented with 0.2% casamino acids and 0.2% glucose **(C)** and analyzed by flow cytometry. The doubling times (Td) [min], relative ratios of cell mass, ori/mass, and initiation mass (Mi) (set MG1655 cells [1] as a standard) are shown in the histogram. Errors represent the standard deviations calculated from at least three independent experiments. **(D)** MG1655, STM701 [Δ*mnmC*], STM702 [Δ*mnmE*], STM703 [Δ*mnmG*], and STM706 [Δm*iaB*] cells were cultivated at 37 °C in LB medium and analyzed by flow cytometry. Errors represent the standard deviations calculated from three independent experiments. **(E–F)** Effect of deletion of *ygfZ* on hydroxyurea resistance. Serial dilutions of MG1655, STM704, and STM706 cells were cultivated at 37 °C for 24h **(E)** or at 30 °C for 48h **(F)** on LB plates containing indicated concentrations (0, 10, or 12.5 mM) of hydroxyurea and a spot assay was performed.

Next, we focused on the role of YgfZ. Consistently with a previous report (Lund et al., 2023), Δ*ygfZ* cells cultivated at 37 °C in LB medium exhibit reduced growth rates (Td = 43 ± 9 min) and impaired initiation (ori/mass = 0.75 ± 0.09, Mi = 1.4 ± 0.1) compared with WT cells (Figure 2C). Δ*ygfZ* cells also exhibited impaired initiation in the defined media, M9 medium supplemented with glucose and casamino acids (Figure 3C) and with glycerol (Supplementary Figure 1A). By contrast, single deletions of several TCA cycle enzymes little affected initiation or growth rates of the cells (Supplementary Figure 1B), consistent with previous reports (Westfall and Levin, 2018). To further address the relationship between YgfZ and the TCA cycle in the regulation of replication initiation, we performed succinate dehydrogenase assays using cells cultivated in LB medium. The results were consistent with those reported in the previous study using *E. coli* cells in early stationary phase (OD_600_~1) (Waller et al., 2010), and demonstrated severe impairment of succinate dehydrogenase activity in exponentially-growing Δ*ygfZ* cells (OD_600_ ~ 0.5) (1.9 ± 0.9 U/mg proteins) compared with that in WT cells (30 ± 3 U/mg proteins) (Figure 2D). In contrast, deletion of *ygfZ* little affected the cellular ATP level in Δ*ygfZ* cells (OD_600_ ~0.2) (83 ± 4%) compared with that in WT cells (88 ± 7%) (Figure 2E), suggesting that that ATP itself is not a direct determinant for regulating cellular ATP-DnaA level. Taken together, these results support the idea that YgfZ functions as a stimulatory factor of replication initiation by mediating the overall metabolic states or processes including TCA cycle with the regulatory systems for replication initiation in a direct or indirect manner under various growth conditions (Figure 2A).

### Initiation regulation by YgfZ is independent of tRNA modification

Given the inhibition of MiaB-dependent tRNA modification (ms^2^i^6^A37) in Δ*ygfZ* cells and its suppression by deleting *mnmE* (Lund et al., 2023; Ote et al., 2006; Waller et al., 2012b), we next examined initiation activity (ori/mass and initiation mass values) in cells deleted in one or more of tRNA modification genes *mnmA, mnmC-E-G*, and *miaB* (Figures 3A–D) (Lund et al., 2023; Waller et al., 2012b; Zhou et al., 2021). Among these enzymes, MnmA and MiaB interact with [Fe-S] cluster. As described in previous studies (Ikeuchi et al., 2006; Nilsson et al., 2017), Δ*mnmA* cells cultivated in LB medium show lower growth rates (Td = 40 ± 6 min) than WT cells (Td = 23 ± 3 min). Flow cytometry analysis revealed that *mnmA* deletion reduced cell mass (Mass = 0.73 ± 0.01) and the *oriC* number per cell was markedly reduced (Figure 3B). As a consequence of these effects on cell division and replication initiation, the initiation profiles of Δ*mnmA* cells were comparable to WT cells (ori/mass = 1.0 ± 0.0, Mi = 1.1 ± 0.2, set WT as a standard) (Figure 3B). When cultivated in M9 supplemented with glucose and casamino acids, basically similar features were observed and the delay in the initiation timing during the cell cycle (ai) was evident in Δ*mnmA* cells (Figure 3C). These results suggest that deletion of *mnmA* alters regulations for cell division and replication initiation. In the Δ*ygfZ* Δ*mnmA* double deletion cells, those parameters were shown as intermediates of those of the two single mutant cells (Figures 3B, C). These results are consistent with the idea that YgfZ and MnmA play stimulating roles for replication initiation directly or indirectly and that MnmA plays a regulatory role also in sustaining the timing of cell division directly or indirectly to maintain the proper cell size.

The initiation parameters of Δ*mnmC*, Δ*mnmE*, and Δ*mnmG* cells were comparable to those of WT cells (Figure 3D). In Δ*miaB* cells, the cell mass was slightly decreased, and the ori/mass was slightly increased with a slight decrease in Mi, suggesting a slight stimulation in the replication initiation. All the mutants preserved the growth rates of cells at the WT level. Given that tRNA modifications (ms^2^i^6^A37 and mcm^5^s^2^U34) are impaired in these mutants, these results indicate that inhibition in replication initiation in Δ*ygfZ* cells can not be attributed to the impaired tRNA modifications.

### Deletion of *ygfZ* confers hydroxyurea resistance

Previous studies have shown that reduced replication initiation can lead to resistance to hydroxyurea, a drug that depletes intracellular dNTP pools by inhibiting ribonucleotide reductase and induces replication fork arrest (Davies et al., 2009; Kasho et al., 2025; Noguchi and Katayama, 2016). For example, Δ*diaA* or Δ*DARS2* mutants, both of which exhibit impaired initiation, display resistance to hydroxyurea. To investigate this phenomenon within the context of the present study, we assessed the hydroxyurea sensitivity of Δ*ygfZ* and Δ*miaB* cells. The results revealed that WT and Δ*miaB* cell growth was sensitive to 12.5 mM hydroxyurea at 30 °C (Figure 3E) and 10 mM hydroxyurea at 37 °C (Figure 3F). In addition, Δ*mnmA* cells showed sensitivity against hydroxyurea, as shown in a previous report indicating reduction in expression of ribonucleotide reductase in Δ*mnmA* cells (Nakayashiki et al., 2013) (Figures 3E, F). By contrast, while Δ*ygfZ* cells showed slower growth even in the absence of hydroxyurea, the addition of hydroxyurea did not appreciably affect their growth rate. Consistent with flow cytometry analysis, the Δ*ygfZ* Δ*mnmA* double deletion cells showed intermediate growth levels in the presence of hydroxyurea. Thus, the observed hydroxyurea resistance of Δ*ygfZ* cells are consistent with the idea of YgfZ-dependent stimulation of replication initiation.

### Deletion of *ygfZ* enhances *datA* function

To clarify how YgfZ stimulates replication initiation, we examined whether deletion of *ygfZ* enhances *datA* function or inhibits *DARS2* function (Figure 1C). Our previous study revealed that deletion of *ygfZ* suppresses the temperature-sensitive growth of *had* (Ts) cells and decreases the cellular level of ATP-DnaA (Ote et al., 2006), suggesting a role for YgfZ in the regulation of *datA* or *DARS2*. We performed flow cytometry to compare the activities of *datA* and *DARS2* in the presence and absence of *ygfZ*.

**Figure 4:**
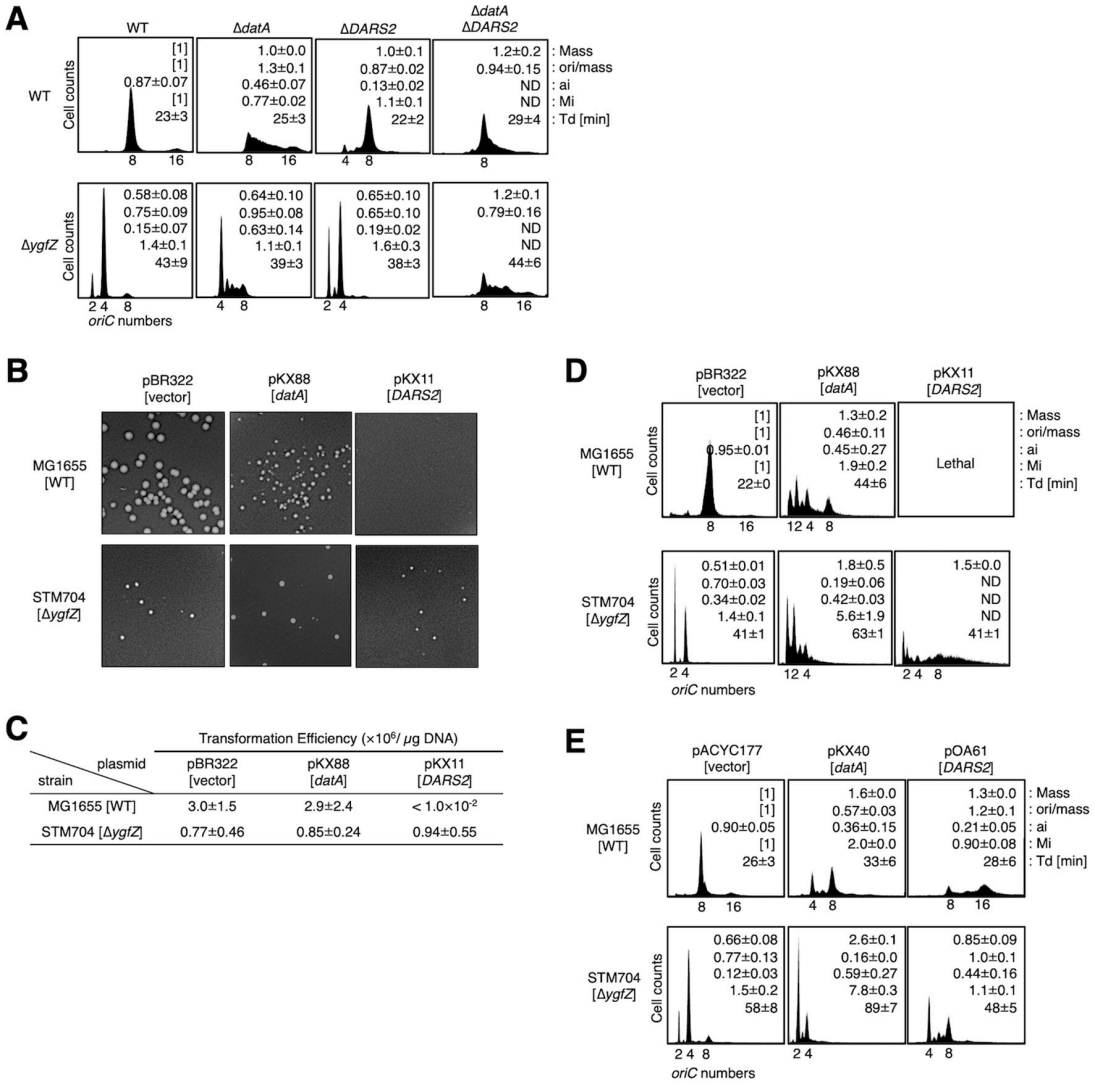
The roles of *YgfZ* in the regulation of *datA* and *DARS2* function. **(A)** MG1655 [WT], SA182 [Δ*datA*], MIT78 [Δ*DARS2*], IKH16 [Δ*datA* Δ*DARS2*], STM704 [Δ*ygfZ*], STM708 [Δ*ygfZ* Δ*datA*], STM707 [Δ*ygfZ* Δ*DARS2*], and IKH17 [Δ*ygfZ* Δ*datA* Δ*DARS2*] cells were cultivated at 37 °C in LB **(A)** medium and analyzed by flow cytometry. The doubling times (Td) [min], relative ratios of cell mass, ori/mass, and initiation mass (Mi) (set MG1655 cells [1] as a standard) are shown in the histogram. Errors represent the standard deviations calculated from three independent experiments. **(B, C)** The plasmids pBR322 [vector], pKX88 [pBR322-datA], or pKX11 [pBR322-*DARS2*] were introduced into MG1655 and STM704 cells and cultivated at 37 °C in LB plate containing 100 μg/mL ampicillin, followed by analysis of colony formation **(B)** and transformation efficiency **(C)**. Two independent experiments were performed, and the mean values and errors are indicated. **(D)** MG1655 and STM704 cells harboring pBR322, pKX88, or pKX11 were cultivated at 37 °C in LB medium containing 50 μg/mL ampicillin and analyzed by flow cytometry. The doubling times (Td) [min], relative ratios of cell mass, ori/mass, and initiation mass (set MG1655 cells harboring pBR322 [1] as a standard) are shown in the histogram. Errors represent the standard deviations calculated from three independent experiments. ND represents ‘not determined'. **(E)** MG1655 and STM704 cells harboring low-copy plasmids pACYC177 [vector], pKX40 [pACYC177-*datA*], or pOA61 [pACYC177-*DARS2*] were cultivated at 37 °C in LB medium containing 50 μg/mL ampicillin and analyzed by flow cytometry. The doubling times (Td) [min], relative ratios of cell mass, ori/mass, and initiation mass (set MG1655 cells harboring pACYC177 [1] as a standard) are shown in the histogram. Two independent experiments were performed, and the mean values and errors are indicated.

Consistent with our previous data (Kasho et al., 2024, 2025), when cultivated at 37 °C in LB medium, SA182 [Δ*datA*] cells showed an increase of initiation activity (ori/mass = 1.3 ± 0.1, Mi = 0.77 ± 0.02), while MIT78 [Δ*DARS2*] showed a decrease (ori/mass = 0.87 ± 0.02, Mi=1.1 ± 0.1), compared with MG1655 [WT] cells (set ori/mass = 1, Mi = 1 as a standard) (Figure 4A). As shown in Figure 2, STM704 [Δ*ygfZ*] cells exhibited a significant decrease of initiation activity (ori/mass = 0.75 ± 0.09, Mi = 1.4 ± 0.1), consistent with the previous data indicating the decrease in DnaA activity in Δ*ygfZ* cells. Furthermore, in the Δ*ygfZ* genetic background, the additional deletion of *datA* [STM708 (Δ*ygfZ* Δ*datA*)] substantially increased the initiation activity (ori/mass = 0.95 ± 0.08, Mi = 1.1 ± 0.1), restoring it to levels comparable to those of MG1655 cells (Figure 4A). Conversely, deletion of *DARS2* in the Δ*ygfZ* background [STM707 (Δ*ygfZ* Δ*DARS2*)] slightly decreased the initiation frequency (ori/mass = 0.65 ± 0.10, Mi = 1.6 ± 0.3) without statistical significance compared with Δ*ygfZ* cells with intact *DARS2* (Figure 4A). Consistent with this, double deletions of *datA* and *DARS2* in WT cells decreased the ori/mass, whereas those in Δ*ygfZ* cells increased it. Also, the comparison of Δ*datA* vs. Δ*datA* Δ*DARS2* also suggested that the effect of *DARS2* deletion was reduced in Δ*ygfZ* cells: i.e., *DARS2* deletion decreased the ori/mass by 0.36 (ori/mass = 1.3 ± 0.1 in Δ*datA*, and ori/mass = 0.94 ± 0.15 in Δ*datA* Δ*DARS2*) in WT cells, whereas only decreased by 0.16 (ori/mass = 0.95 ± 0.08 in Δ*ygfZ* Δ*datA*, and ori/mass = 0.76 ± 0.16 in Δ*ygfZ* Δ*datA* Δ*DARS2*) in Δ*ygfZ* cells, supporting our conclusion that the *DARS2* function is limited in Δ*ygfZ* cells.

Further supportive evidence of the above came from phenotypic analyses of cells carrying multicopy *datA* or *DARS2*. We transformed MG1655 [WT] or STM706 [Δ*ygfZ*] cells with the pBR322-based plasmids pKX88 containing *datA* or pKX11 containing *DARS2* and assessed colony formation and transformation efficiency using pBR322 as a control. As described previously (Fujimitsu et al., 2009; Kasho et al., 2014), introduction of pKX11 [pBR322-*DARS2*] severely inhibited colony formation in MG1655 cells due to excessive initiation of chromosome replication (Figures 4B, C). Strikingly, this inhibition was totally alleviated in the Δ*ygfZ* strain, indicating that initiation activity was significantly downregulated upon deletion of *ygfZ*. This observation suggests the idea that *DARS2* function is limited in Δ*ygfZ* cells. In these experiments, the deletion of *ygfZ* had little effect on transformation efficiency of cells bearing pKX88 (Figures 4B, C). To further assess the activity of replication initiation, we performed flow cytometry. As described previously (Kasho et al., 2017), introduction of pKX88 [*datA*] severely inhibits initiation in MG1655 cells (Figure 4D). This is consistent with the reduced growth rate of cells bearing pKX88 (Figures 4B, D). Notably, consistent with our findings in Figure 4A, initiation activity was further inhibited in Δ*ygfZ* cells bearing pKX88 (Figure 4D), demonstrating that the combination of the *ygfZ* deletion and multicopy *datA* profoundly impairs initiation. Conversely, Δ*ygfZ* cells bearing pKX11 [pBR322-*DARS2*] were viable but moderately exhibited excessive initiations (Figure 4D), supporting the conclusion that *DARS2* activity is decreased in Δ*ygfZ* cells.

The results obtained using pACYC177-based plasmids (Figure 4E) were consistent with those obtained above using pBR322-based plasmids. The moderate copy number of pACYC177 allows MG1655 cells bearing pOA61 [pACYC177-*DARS2*] to grow (Fujimitsu et al., 2009; Kasho et al., 2014, 2025). Replication initiation was inhibited in MG1655 cells bearing pKX40 [pACYC177-*datA*] and the deletion of *ygfZ* further exacerbated the inhibition. In contrast, excessive initiations caused by pOA61 was moderate in in Δ*ygfZ* cells, compared to those in MG1655 cells. Taken together, YgfZ profoundly downregulates the *datA* function while it supports the *DARS2* function.

### Deletion of *ygfZ* broadly increases genomic IHF binding with the initiation stage-specific oscillation at key loci

**Figure 5:**
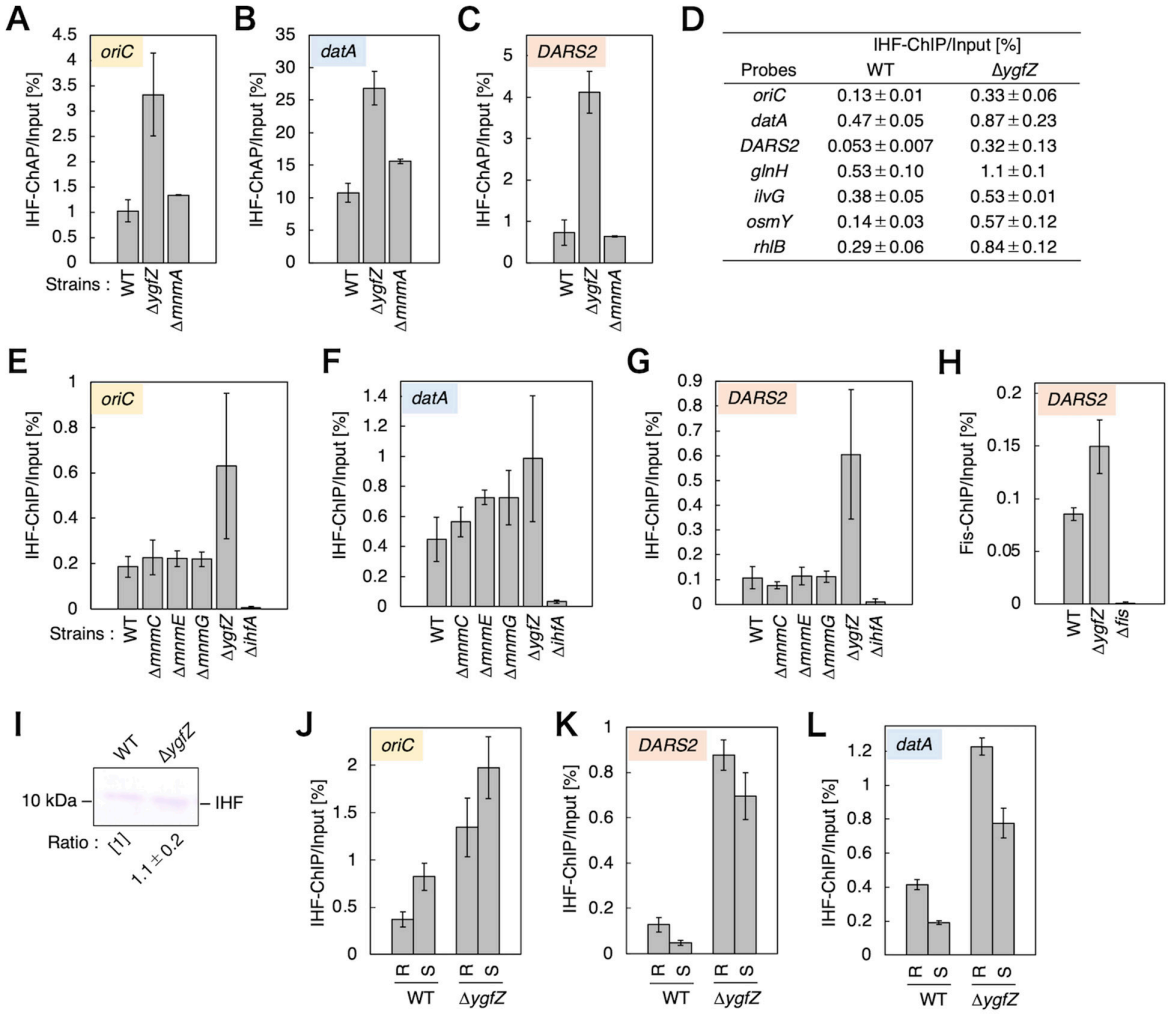
The roles of *YgfZ* in the regulation of genomic IHF binding. **(A–C)** IHF ChAP-qPCR. KX200 [*ihfA-cHis*^12^], KX283 [*ihfA-cHis*^12^ Δ*ygfZ*], and KX293 [*ihfA-cHis*^12^ Δ*mnmA*] cells were cultivated at 37 °C in LB medium, followed by ChAP-qPCR. The ChAP/Input for *ylcC* (%) was used as a background control for non-specific IHF binding and was subtracted from the ChAP/Input for *oriC* **(A)**, *datA* **(B)**, and *DARS2* **(C)**. Error bars represent the standard deviations calculated from at least 3 independent experiments. Based on the Student's *t*-test, the comparisons of ChAP/Input for *oriC* in Δ*ygfZ* cells vs. that of WT (*p* < 0.002), ChAP/Input for *datA* in Δ*ygfZ* cells vs. that of WT (*p* < 0.0002), ChAP/Input for datA in Δ*mnmA* cells vs. that of WT (*p* < 0.007), and ChAP/Input for *DARS2* in Δ*ygfZ* cells vs. that of WT (*p* < 0.00002) are statistically significant, whereas others were not. **(D–G)** IHF ChIP-qPCR. **(D)** MG1655 [WT] and STM704 [Δ*ygfZ*] cells were cultivated at 37 °C in LB medium, followed by ChIP-qPCR analysis. The ChIP/Input for *ylcC* (%) was used as a background control for non-specific IHF binding and was subtracted from the ChIP/Input for *oriC, datA, DARS2, glnH, ilvG, osmY*, and *rhlB*. Error bars represent the standard deviations calculated from 2 independent experiments. **(E–G)** MG1655, STM701 [Δ*mnmC*], STM702 [Δ*mnmE*], STM703 [Δ*mnmG*], STM704, and KMG-5 [Δ*ihfA*] cells were cultivated at 37 °C in LB medium, followed by ChIP-qPCR analysis. The ChIP/Input (%) for *oriC* **(E)**, *datA* **(F)**, and *DARS2* **(G)** were determined. Error bars represent the standard deviations calculated from 2 independent experiments. Based on the Student's *t*-test, the comparisons of ChAP/Input for *oriC* in Δ*ygfZ* cells vs. that of WT (*p* < 0.03), ChAP/Input for *datA* in Δ*ygfZ* cells vs. that of WT (*p* < 0.007), ChAP/Input for *DARS2* in Δ*ygfZ* cells vs. that of WT (*p* < 0.008), as well as the comparison of WT vs. Δ*ihfA* cells are statistically significant, whereas others were not. **(H)** Fis ChIP-qPCR. MG1655 and STM704 cells were cultivated at 37 °C in LB medium, followed by ChIP-qPCR analysis using anti-Fis antiserum. Error bars represent the standard deviations calculated from 2 independent experiments. **(I)** IHF immunoblotting. The relative amount of IHF in STM704 [Δ*ygfZ*] cells were compared with that in MG1655 [WT] cells, set [1] as a standard. Error represents the standard deviations calculated from three independent experiments. **(J-L)** IHF ChIP-qPCR using *dnaC2* cells. KYA018 [*dnaC2*] and STM715 [*dnaC2* Δ*ygfZ*] cells were cultivated at 30 °C in LB medium (R; Random) and transferred to 38 °C and incubated for 90 min (S; Synchronized), followed by ChIP-qPCR analysis. The ChIP/Input (%) for *oriC* **(J)**, *datA* **(K)**, and *DARS2* **(L)** were determined. Error bars represent the standard deviations calculated from three independent experiments. Based on the Student's *t*-test, the comparisons of ChAP/Input for oriC in Δ*ygfZ-S* vs. that of Δ*ygfZ-R* (*p* < 0.002), ChAP/Input for datA in Δ*ygfZ-S* vs. that of Δ*ygfZ-R* (*p* < 0.006), ChAP/Input for *DARS2* in Δ*ygfZ-S* vs. that of Δ*ygfZ-R* (*p* < 0.008), as well as the comparison of WT vs. Δ*ygfZ*, or the comparison of WT-S vs. WT-R are statistically significant, whereas others were not.

To investigate whether deletion of *ygfZ* impacts IHF binding at key loci such as *oriC, datA*, and *DARS2* (Figure 1A), we performed ChAP-qPCR experiments using KX200 [*ihfA-cHis*_12_], KX283 [*ihfA-cHis*_12_ Δ*ygfZ*], and KX293 [*ihfA-cHis*_12_ Δ*mnmA*] cells. Compared with the ChAP/input values in WT cells, deletion of *ygfZ* broadly increased IHF binding across all tested IBSs (Figures 5A–C; Supplementary Figure S2A). By contrast, deletion of *mnmA* moderately increased local *datA*-IHF binding (Figure 5B; Supplementary Figure 2A). The elevated *datA*-IHF-binding levels in Δ*ygfZ* and Δ*mnmA* cells are consistent with the hypothesis that *datA* function is enhanced in these mutants.

The global increase in IHF binding in Δ*ygfZ* cells was further corroborated by ChIP-qPCR experiments employing anti-IHF antibody (Kasho et al., 2014). Besides *oriC, datA*, and *DARS2* (Kasho et al., 2024), IHF is known to bind to other IBSs in *glnH, ilvG, osmY*, and *rhlB* (Figure 5D; Supplementary Figure 2B). Strikingly, deletion of *ygfZ* broadly increased IHF binding to all tested IBSs with the exception of that in the *ilvG*, relative to the ChIP/input values in WT cells. To rule out the possibility that this increase in IHF binding in Δ*ygfZ* cells was simply a consequence of retarded cell growth, we performed IHF ChIP-qPCR experiments using WT cells cultivated in different types of growth media (Td: 23 ± 2 min in LB, 76 ± 13 min in M9 medium supplemented with 0.2% glucose, and 137 ± 25 min in M9 medium supplemented with 0.6% glycerol) (Supplementary Figure 2C). Crucially, IHF binding at *datA* and *DARS2* regions did not increase with growth retardation (Supplementary Figure 2D). At *oriC*, IHF binding was significantly increased only for cells growing in M9 medium supplemented with 0.6% glycerol, which could be related to elongated duration of the pre-initiation stage (Supplementary Figure 2B). Furthermore, deletion of *lpd* as well as *mnmC, mnmE*, or *mnmG* had a minimal effect on IHF binding (Figures 5E–G; Supplementary Figure 2E), suggesting that genome-wide stimulation of IHF binding is a specific consequence of the *ygfZ* deletion. Based on our previous findings that DNA supercoiling stimulates IHF binding at *datA* and *DARS2* loci, we have analyzed the structure of plasmid DNA purified from Δ*ygfZ* cells (Supplementary Figure 3). Deletion of *ygfZ* decreases DNA supercoiling in the presence or absence of gyrase inhibitor novobiocin at a sublethal level, which is likely an indirect consequence by the excessive IHF binding and the resulting change in DNA topology.

Additional ChIP-qPCR experiments using anti-Fis antibody revealed that *DARS2*-Fis binding was also higher in Δ*ygfZ* cells than in WT cells (Figure 5H). The limited activity of *DARS2* in Δ*ygfZ* cells might therefore be the consequence of the presence of another regulatory mechanism functioning independently of IHF and Fis. To rule out another possibility that the increase in IHF binding in Δ*ygfZ* cells was simply a consequence of increased IHF protein expression, we performed western blotting experiments and confirmed that the expression level of IHF protein was unaffected by deletion of *ygfZ, mnmC, mnmE*, or *mnmG* (Figure 5I), suggesting the importance of IHF dissociation pathways by which YgfZ globally contributes to the control of the basal level of genomic IHF binding.

To further analyze the requirement for YgfZ for the regulation of cell cycle-dependent IHF binding at *oriC, datA*, and *DARS2*, we utilized temperature-sensitive *dnaC2* cells, which permit cell cycle synchronization in LB medium (Figures 5J–L)(Withers and Bernander, 1998). The *dnaC2* mutation specifically inhibits replication initiation at *oriC* at restrictive temperatures (37-42 °C), enabling cell cycle synchronization at the initiation stage by incubating cells at 37 °C for 80 min (S; Synchronized). This *dnaC2*-based synchronization system was previously successful in identifying the cell cycle-coordinated IHF-binding/dissociation pattern at *oriC, datA*, and *DARS2* (Kasho et al., 2014; Kasho and Katayama, 2013). Consistently, in KYA018 [*dnaC2*] cells, IHF binding at *oriC* was increased (ChIP/Input = R:0.37 ± 0.08% vs. S:0.82 ± 0.15%) and IHF binding at *datA* and *DARS2* was decreased (*datA* R:0.42 ± 0.03% vs. S:0.19 ± 0.01%; *DARS2* R:0.13 ± 0.03% vs. S:0.048 ± 0.010%) at the initiation stage (S), compared with those in a random culture sample (R; Random). In the random culture samples of STM715 [*dnaC2* Δ*ygfZ*] cells, the overall levels of IHF binding at *oriC, datA*, and *DARS2* were increased (Figures 5J–L), consistent with the data obtained using STM704 [Δ*ygfZ*] cells (Figures 5A–C). Notably, the oscillation of IHF binding persisted even in Δ*ygfZ* cells (Figures 5J–L): compared with IHF binding in a random culture sample (R), IHF binding at *oriC* was increased (R:1.3 ± 0.3% vs. S:2.0 ± 0.3%) and IHF binding at *datA* and *DARS2* was decreased (*datA* R:1.2 ± 0.1% vs. S:0.78 ± 0.09%; *DARS2* R:0.88 ± 0.07% vs. S:0.70 ± 0.10%) at the initiation stage (S). These results suggest that YgfZ is crucial for the regulation of the basal level of genomic IHF binding in coordination with cellular growth conditions, while the cell cycle-coordinated oscillation in IHF binding at the specific loci can operate independently of YgfZ.

### Whole genome analysis reveals specific effects of YgfZ in IHF binding regulation

**Figure 6:**
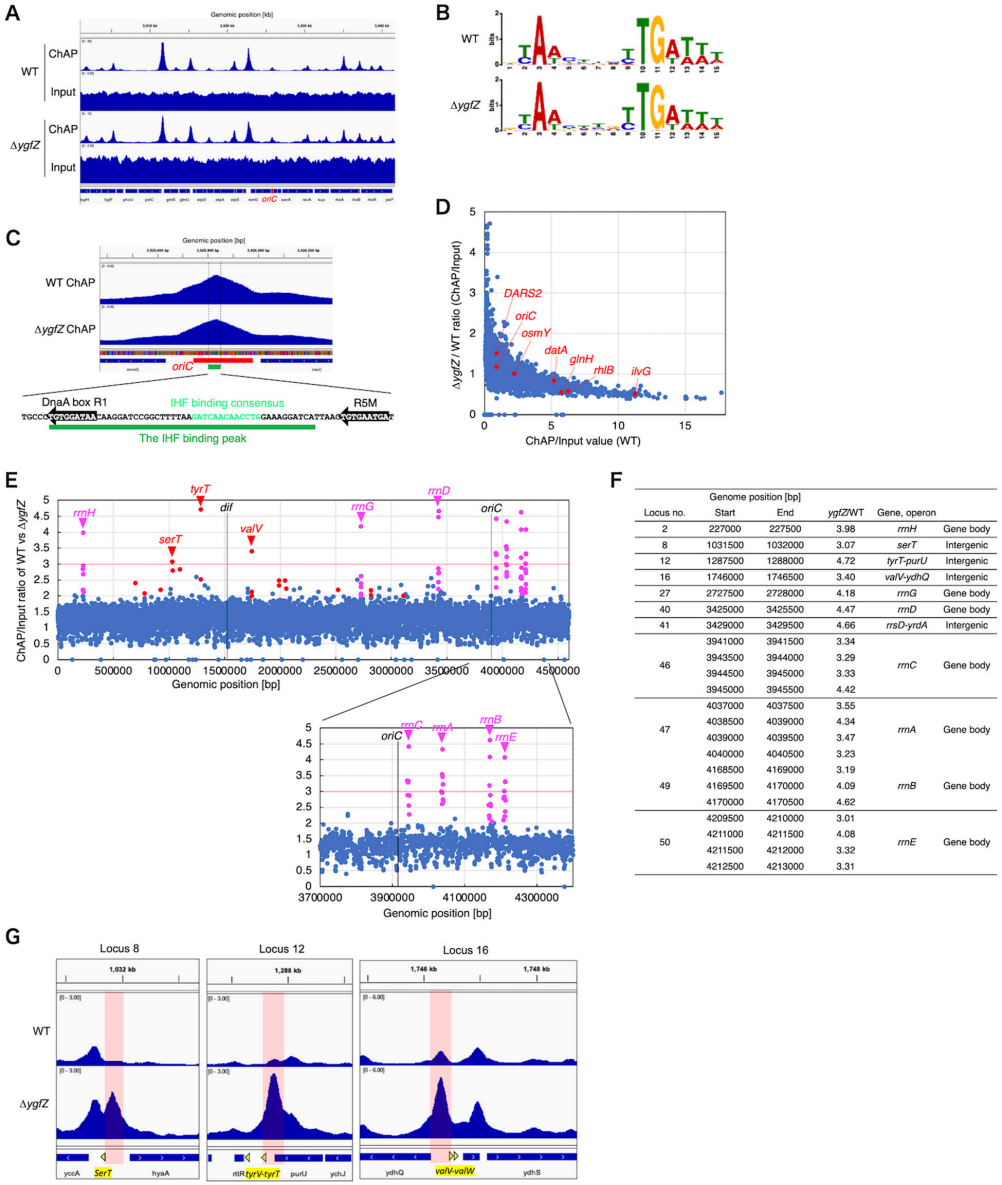
Overall IHF-binding profile in Δ*ygfZ* cells. **(A)** Panels indicate the IHF-binding profiles in ChAP samples and the Input read profiles obtained from the same WT and Δ*ygfZ* samples as used in Figures 5A-C. Read depth of ChAP samples indicates the strength of IHF binding at each genomic loci. To show the ChAP result, the vertical scale was altered 0.5-fold in the Δ*ygfZ* dataset relative to the WT dataset to align the major IHF-binding peaks to similar heights. **(B)** Logos indicating consensus sequences estimated from the top 500 highest IHF-binding peaks in the WT and Δ*ygfZ* ChAP dataset. On the right side of the Logos, datasets and the numbers of highest IHF-binding peaks used to compute the Logos are indicated. **(C)** Extension of Figure 6A at the oriC locus. The upper panel indicates IHF-binding peaks observed in the WT and Δ*ygfZ* ChAP dataset. At the bottom of this Figure, the location of the *oriC* (245 bp) and the highest IHF-binding peak (10-bp window) observed at the *oriC* locus are indicated. The lower panels indicate the DNA sequence of the highest IHF-binding peak, as well as *DnaA* boxes R1, R5M, and IBS. **(D)** Mapping of the changes at each IHF-binding site between WT and Δ*ygfZ* ChAP datasets. The y-axis of graph indicates the ratios of Δ*ygfZ* ChAP/Input values to WT ChAP/Input values, while the x-axis indicates WT ChAP/Input values. **(E)** Mapping the genome-wide changes in the IHF-binding pattern between WT and Δ*ygfZ* ChAP datasets. The y-axis of graph indicates the calculated ratios of Δ*ygfZ* ChAP/Input values of each 500-bp window of read depth to WT ChAP/Input values, while the x-axis shows the genomic positions [bp]. The pink and red arrowheads indicate seven *rrl* gene bodies (*rrlA, rrlB, rrlC, rrlD, rrlE, rrlG*, and *rrlH*) encoding 23S rRNA and tRNA gene clusters (*ileV-alaV, gltW, ileU-alaU, gltV, ileT-alaT, gltV*, and *gltT*) and the intergenic regions adjacent to three other tRNA genes (*serT, tyrT*, and *valV*), respectively, where the most significant increase in the WT/Δ*ygfZ* ratio was observed, as listed in **(F)**. **(G)** The IHF-binding patterns at three tRNA genes (serT, tyrT, and valV) are highlighted.

To gain a comprehensive understanding of how YgfZ impacts genome-wide IHF binding, we performed IHF ChAP-seq analysis using the same ChAP and Input samples from KX200 [*ihfA-cHis12*] and KX283 [*ihfA-cHis12* Δ*ygfZ*] cells as those used in Figures 5A–C (Figure 6A). The ChAP-seq results for WT and Δ*ygfZ* samples robustly identified known IBSs previously determined by *in vitro* DNase I footprinting and *in vivo* GeF-seq (Genome footprinting with high-throughput sequencing) experiments (Chumsakul et al., 2018; Kasho et al., 2021), including those at *oriC, datA, DARS2, ilvG, osmY*, and *glnH*. Also, MEME suite predicted that the consensus sequences predicted from the top 500 IHF-binding peaks with highest read depth in both the WT and Δ*ygfZ* ChAP dataset were largely identical (WT: YAWnnnnYTGAWWW; Δ*ygfZ*: YAWnnnnYTGAWWW, where Y is T or C, W is A or T, and n is any nucleotide) (Figure 6B). This consensus sequence precisely corresponds to the representative 13-mer IHF-binding consensus TAAnnnnTTGATW (Aeling et al., 2006). For example, the IHF-binding peaks that mapped within a 10 bp window at the *oriC* and *datA* loci precisely overlapped with the 13-mer IHF-binding consensus sequence (Figure 6C; Supplementary Figure 4A), consistent with previous studies. The IHF binding signal mapped at the *DARS2* locus was overlapped with IBS1-2, consistent also with previous studies. In addition, we observed a stronger binding peak at low affinity IBS just outside *DARS2* (Supplementary Figure 4B) (Kasho et al., 2014), which suggests the possibilities that the observed increase of IHF binding in Δ*ygfZ* cells in our ChAP/ChIP-qPCR experiment (Figure 5) could be the combined effect of *DARS2* IBS1-2 and this additional IBS and that the additional IHF binding might cause the formation of abortive complexes and be responsible for the inactivation of *DARS2* in Δ*ygfZ* cells (Figure 4).

To remove the copy number effects and pinpoint specific changes in the IHF-binding pattern between WT and Δ*ygfZ* ChAP datasets (Supplementary Figure 4D), we calculated the ChAP/Input values for each 500-bp window of read depth and then determined the ratio of Δ*ygfZ* ChAP/Input values to those of the WT (Figure 6D). Because the total reads are fixed in each NGS samples, the ChAP/Input ratio at each genomic loci are enriched or reduced in a manner dependent on their IHF binding affinity (x-axis in Figure 6D). When the Δ*ygfZ*/WT ChAP/Input ratio was mapped against the WT ChAP/Input dataset (y-axis in Figure 6D), it was apparent that the global patterns of IHF-binding peaks were remarkably similar between WT and Δ*ygfZ* ChAP cell samples. However, it is noticeable that in Δ*ygfZ* cells, IHF binding was particularly promoted at regions where IHF binding is inherently weak in WT cells. In contrast, the Δ*ygfZ*/WT ChAP/Input ratio was relatively low at regions where IHF binding is inherently high in WT cells (e.g., *ilvG, rhlB*, and *glnH*). Taken together with the fact that the total reads of WT and Δ*ygfZ* ChAP datasets are comparable (WT: 22,210,158 reads and Δ*ygfZ*: 22,537,532 reads), the IHF ChAP-seq data indicate that deletion of *ygfZ* increases IHF binding at global genomic sites, consistent with the ChAP/ChIP-qPCR data (Figure 5). As explained above, the increase is particularly noticeable at low affinity sites, which limits the number of sequence reads for other sites, resulting in the relative decrease of the Δ*ygfZ*/WT ChAP/Input ratio of high affinity IHF binding sites.

Mapping of the Δ*ygfZ*/WT ChAP/Input ratios to genomic positions revealed that IHF binding was typically enhanced at the regions adjacent to tRNA genes (21 out of 51 loci; Supplementary Figures 4, 5) and strongly promoted (Δ*ygfZ*/WT>3) at seven *rrl* gene bodies (*rrlA, rrlB, rrlC, rrlD, rrlE, rrlG*, and *rrlH*) encoding 23S rRNA and tRNA genes located in *rrn* operons (*rrnA, rrnB, rrnC, rrnD, rrnE, rrnG*, and *rrnH*), as well as at intergenic regions adjacent to the transcriptional promoters of three other tRNA genes (*serT, tyrT*, and *valV*) (Figures 6E–G; Supplementary Figures 5, 6). The observed increase in IHF binding was not confined to gene bodies but was also seen at intergenic regions, regardless of transcriptional direction (Supplementary Figure S5). This finding suggests that the widespread enhancement of IHF binding is a consequence of the combined effect of global change in chromosome structure and localized change in transcriptional dynamics.

## Discussion

**Figure 7:**
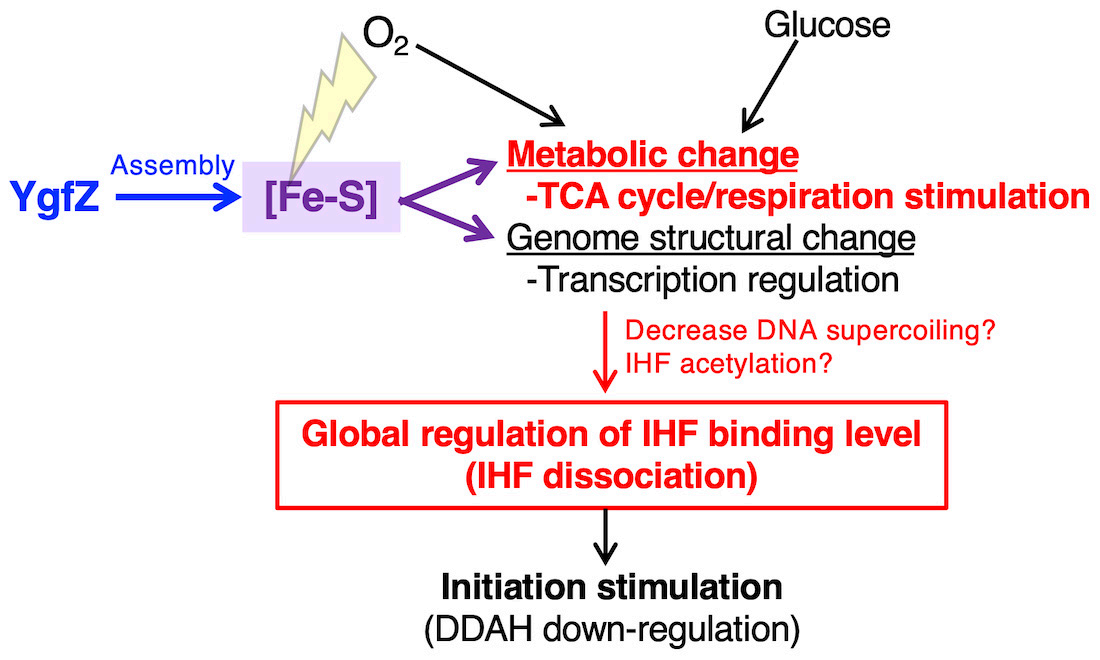
A novel link between [Fe-S] maintenance and replication initiation. Regulatory model of replication initiation and genomic IHF binding. Under aerobic and nutrient-rich environments, [Fe-S] clusters stimulate glucose metabolism, resulting in the stimulation of replication initiation and regulation of the basal IHF-binding level. Given that [Fe-S] clusters control the TCA cycle, the first pathway likely represents a control system coupled with glucose metabolism. In this context, deletion of *ygfZ* induces the instability of [Fe-S] clusters in the presence of oxidative stress. Furthermore, analysis of IHF dynamics revealed that *YgfZ* predominantly modulate the basal level of IHF binding across the whole genome via multiple pathways, possibly by alteration of DNA topoisomerase activity or by IHF acetylation. Taken togethr, we propose that *YgfZ* is important in coordination of these multifaceted regulatory systems to optimize replication initiation and proliferation, thereby enabling adaptation to diverse environmental conditions.

This study establishes a novel role for YgfZ as a global regulator of IHF binding and a novel link between the YgfZ function and initiation of chromosome replication via downregulation for the *datA*-IHF binding (Figure 7). We provide the first direct evidence that the [Fe-S] assembly factor YgfZ, which stimulates various [Fe-S]-dependent enzymes such as succinate dehydrogenase, fumarase, and DMSO reductase (anaerobic electron transfer enzyme) (Waller et al., 2010), drastically affects the global binding dynamics of IHF across the *E. coli* chromosome including the key loci governing DnaA activity, namely the IBSs in *datA* and *DARS2*. This finding is particularly significant as it likely connects YgfZ-dependent metabolic states to the physical state of the genome (Figure 7). Based on the crucial role for YgfZ in stimulation of [Fe-S]-binding factors, specific factors with [Fe-S] cluster might affect IHF binding dynamics directly or indirectly.

Using a combination of ChAP/ChIP-qPCR and ChAP-seq, this study revealed that deletion of *ygfZ* significantly and broadly increases genomic IHF-DNA binding including *oriC, datA*, and *DARS2* without altering the canonical IHF binding consensus. Despite the increase in basal IHF levels in Δ*ygfZ* cells, the fundamental cell cycle-dependent oscillation of IHF binding at *oriC, datA* and *DARS2* persists, indicating the existence of a two-tiered regulatory system: the first one, dependent on YgfZ, that sets the global, metabolic-driven IHF binding level, and the second, YgfZ-independent mechanism that choreographs the precise, timely IHF dynamics required for replication initiation.

Our findings further dissect the intricate regulatory network controlling the DnaA cycle. We demonstrate that deletion of *ygfZ* significantly enhances the DnaA-inactivating function of the *datA* locus while simultaneously limiting the DnaA-reactivating function of the *DARS2* locus. This provides a clear mechanistic explanation for our previous observation that Δ*ygfZ* suppresses the growth defects and reduces aberrantly high ATP-DnaA levels in temperature-sensitive *hda* mutants. In *datA*, the increase of IHF binding directly enhances the DnaA-inactivating function. Unlike this case, *DARS2* activity might be inhibited by excessive IHF binding, consistent with previous data showing several low-affinity IBSs in *DARS2* region and the idea that excessive IHF binding to these sites downregulates the DnaA-reactivating function. We also show that the [Fe-S]-binding protein MnmA, which is involved in tRNA modification and iron uptake (Leimkühler, 2025; Olivieri et al., 2024), partly regulates local *datA*-IHF binding in a manner independently of YgfZ. The limited role of MnmA could be due to its interaction with specific [Fe-S]-binding factors affecting *datA*-IHF binding directly or indirectly. Additionally, the minimal effects of *mnmCEG* or *miaB* deletion on initiation activity suggest that YgfZ-dependent IHF regulation is independent of tRNA mcm^5^s^2^U34 and ms^2^i^6^A37 modifications (Figure 3C). The minimal impact of *lpd* deletion on IHF binding further suggests the existence of YgfZ-dependent specific pathways to regulate genomic IHF dynamics in coordination with the growth environments (Figure 7). This work thus establishes a model that YgfZ participates in a regulatory system for modulating the genome-wide IHF binding states in a manner dependent on glucose metabolism, but not tRNA modification, thereby coordinating the timing of replication initiation with growth conditions via the DnaA cycle. However, the physiological relevance of YgfZ-mediated regulation remains to be elucidated in various growth conditions and warrants further investigation.

Our discovery has broad implications for understanding cellular integrity and evolution. About the role of YgfZ on global regulation of IHF binding, we propose a few mechanistic models where YgfZ's role in [Fe-S] assembly is indirectly linked to genome architecture. A possible mechanism could involve the modulation of polyamines like spermidine. Spermidine is linked to the TCA cycle and DNA gyrase activity (Deng et al., 2023), and its accumulation is known to stimulate genome-wide binding of IHF and Fis (Kumar et al., 2022). In addition, we do not exclude a possibility that unlike plasmid DNA (Supplementary Figure S3), genome DNA could be enhanced in forming negative supercoils to stimulate IHF binding.

Another possible model is that post-translational modifications (PTMs) could serve as a link (AbouElfetouh et al., 2015; Dilweg and Dame, 2018). Specifically at stationary phase, the acetylation of key lysine residue K178 for ATP binding inhibits the *oriC*-DnaA complex formation (Zhang et al., 2016), which might not be involved in YgfZ-dependent regulation of replication initiation at exponentially phase. The acetylation of key lysine residues on IHF could neutralize its positive charge, reducing its DNA binding activity (Dilweg and Dame, 2018), yet the regulatory role of IHF regulation in genome structuring remains elusive. Non-enzymatic protein acetylation is catalyzed by acetyl-phosphate from acetate overflow pathway (Wolfe, 2005), suggesting that PTMs may monitor the cellular metabolic state. The acetate overflow pathway produces acetyl-phosphate from acetyl-CoA when activity of TCA cycle is relatively decreased (Figure 2A). Alternatively, enzymatic protein acetylation requires the NAD+-dependent sirtuin-type deacetylase CobB (AbouElfetouh et al., 2015). Because NAD+ is converted into NADH during TCA cycle, it's possible that impaired TCA cycle activity stimulates IHF acetylation. These types of metabolic-driven PTM are well-documented for eukaryotic histones (Murata et al., 2019), where it plays a conserved role in epigenetic regulation of cell proliferation (Covarrubias et al., 2021). Analysis on these and other possible mechanisms should be important future study.

About the role of YgfZ on local regulation of IHF binding, we also observed a striking local enrichment at highly transcribed 7 *rrn* operons and specific tRNA loci in addition to a robust, genome-wide increase in IHF binding (Figures 6E–G; Supplementary Figures 4, 5) (Cabrera et al., 2009). While the regulatory role of IHF on tRNA expression remains unknown, overall level of tRNA expression is known to be regulated in a manner coordinated with growth rate. And IHF dissociation from *datA* is supported by transcriptional read-through derived from tRNA (*glyV-glyX-glyY*) operon (Kasho et al., 2024), suggesting that, in addition to the global regulation of basal IHF binding, YgfZ locally promotes IHF dissociation, or prevents its re-binding through a mechanism in coordination with tRNA gene transcription (Waller et al., 2010; Zhou et al., 2021).

As such, our findings provide a novel link between the YgfZ functions and genome-wide IHF binding for the stimulation of replication initiation, despite the current paucity of information on the relationship between chromosome structure and [Fe-S] homeostasis. In ancient, anaerobic environments, life likely acquired [Fe-S] clusters as essential co-factors for electron transfer. While relatively stable under primordial anoxic conditions, their stability was compromised as life adapted to aerobic respiration. From an evolutionary perspective, the widespread conservation of YgfZ/IBA57 family proteins highlights their proviral roles, not only in the activation of the TCA cycle but has also in other diverse functions, which these proteins acquired throughout evolution (Mühlenhoff et al., 2022; Waller et al., 2012a, 2010). While ancient life forms may have had highly condensed chromosomes for protection in harsh (high temperature, salt, pH), oxygen-free environments, the evolution of the YgfZ/IBA57 system in aerobic conditions likely stabilized [Fe-S] clusters and enabled chromosomes to become more relaxed. This dramatic improvement in DNA accessibility to RNA polymerases and other regulatory factors, such as NAPs, would have allowed for more flexible and efficient switching between binding and dissociation states. Ultimately, this facilitated more efficient vital biological reactions, including replication initiation and genome maintenance, supporting the flourishing of life in an oxygen-rich world.

## Data Availability

The datasets presented in this study can be found in online repositories. Genome sequencing data were deposited in the DDBJ Sequence Read Archive (DRA) under BioProject number PRJDB40309 and accession numbers DRA026351 (KX200 [WT]#1, #2 ChAP and Input datasets), DRA026352 (KX283 [DygfZ]#1, #2 ChAP and Input datasets), and DRA026353 (KX283 [DygfZ]#3, #4 ChAP and Input datasets).
