# Supplementary material for: The iron-sulfur accelerator YgfZ modulates genome-wide IHF-binding dynamics to regulate replication initiation in Escherichia coli
Source: Front Microbiol. 2026 Mar 17;17:1781011. doi: 10.3389/fmicb.2026.1781011 (PMC13036160; doi:10.3389/fmicb.2026.1781011)
Supplement: Supplementary file 1 [file Data_Sheet_1.pdf]

Supplementary Material

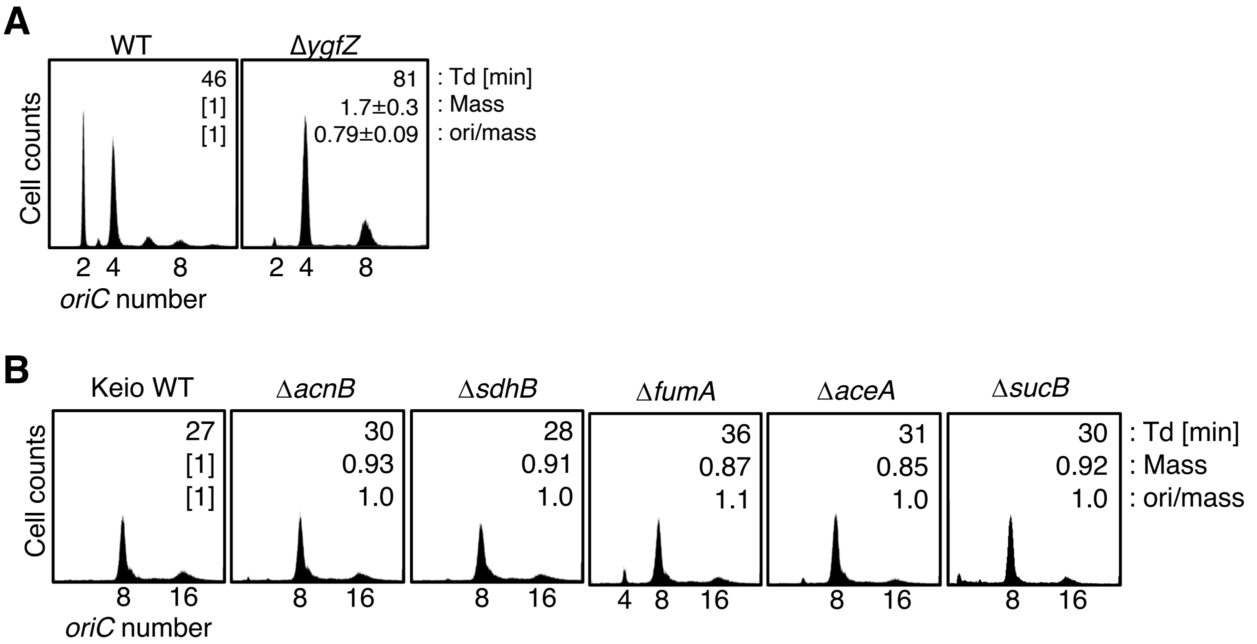

SUPPLEMENTARY FIGURE S1. Effect of TCA enzyme deletion on replication initiation. (A) The replication initiation profiles of MG1655 [WT] and STM704 [Δ*ygfZ*] cells cultivated at 37°C in M9 media supplemented with 0.2% casamino acids and 0.6% glycerol are indicated. The doubling times (Td) [min], relative ratios of cell mass and ori/mass ratios (set MG1655 cells [1] as a standard) are shown in the histogram. (B) The replication initiation profiles of single deletion mutants of each TCA cycle enzymes cultivated at 37°C in LB medium.

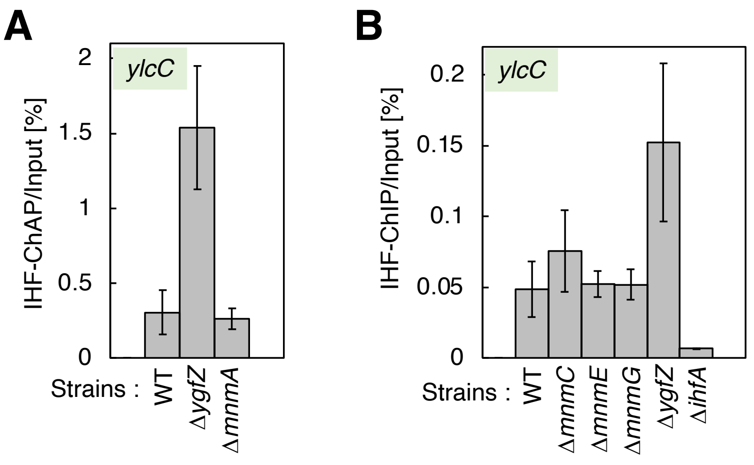

C

Strains	Medium	Doubling time [min]
WT	LB	23 ± 2
WT	M9+0.2% Glc	76 ± 13
WT	M9+0.6% Gly	137 ± 25
ΔygfZ	LB	44 ± 5

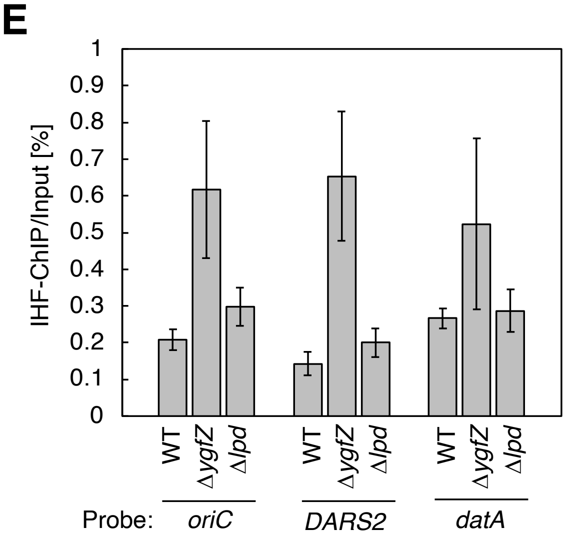

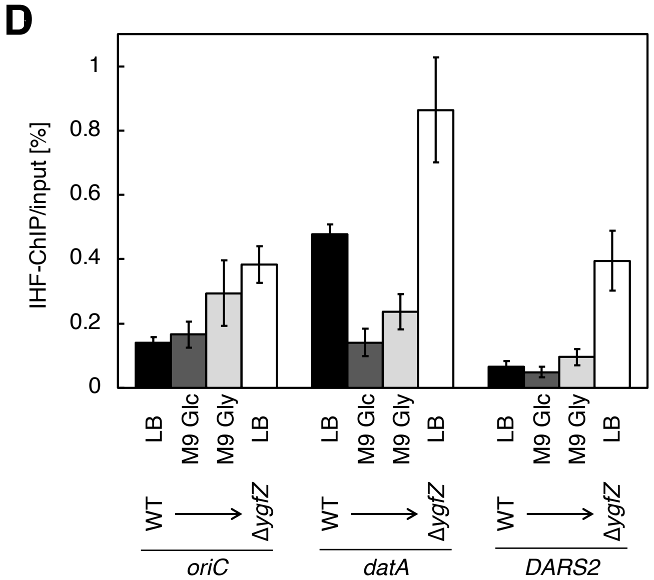

SUPPLEMENTARY FIGURE S2. IHF binding profiles under various growth conditions with different growth rates. (A) IHF ChAP-qPCR. KX200 [*ihfA-cHis12*], KX283 [*ihfA-cHis12* Δ*ygfZ*], and KX293 [*ihfA-cHis12* Δ*mnmA*] cells were cultivated at 37°C in LB medium, followed by ChAP-qPCR. The ChAP/Input for *ylcC* (%) was calculated as a background control for non-specific IHF binding. Error bars represent the standard deviations calculated from at least 3 independent experiments. (B) IHF ChIP-qPCR. MG1655 [WT], STM701 [Δ*mnmC*], STM702 [Δ*mnmE*], STM703 [Δ*mnmG*], STM704 [Δ*ygfZ*], and KMG-5 [Δ*ihfA*] cells were cultivated at 37°C in LB medium, followed by ChIP-qPCR analysis. The ChIP/Input (%) for *ylcC* were calculated. Error bars represent the standard

deviations calculated from 3 independent experiments. (C) The doubling time (Td [min]) and (D) IHF binding profiles at *oriC*, *datA* and *DARS2* loci in MG1655 [WT] and STM704 [Δ*ygfZ*] cells cultivated at 37°C in LB or M9 media supplemented with 0.2% glucose or 0.6% glycerol (without casamino acids) are indicated. The ChIP/Input for *ylcC* (%) was used as a background control for non-specific IHF binding and was subtracted from the ChIP/ Input for *oriC*, *datA*, *DARS2*. Error bars represent the standard deviations calculated from two independent experiments. (E) MG1655, STM704, and KX79 [Δ*lpd*] cells were cultivated at 37°C in LB medium, followed by ChIP-qPCR analysis. Error bars represent the standard deviations calculated from two independent experiments.

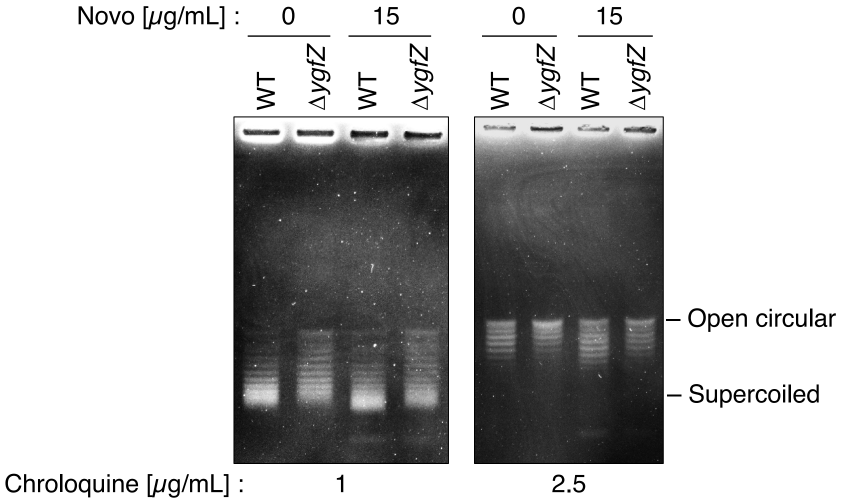

SUPPLEMENTARY FIGURE S3. Effect of *ygfZ* deletion on plasmid DNA supercoiling. The pBR322 DNA was purified from MG1655 [WT] and STM704 [Δ*ygfZ*] cells cultivated at 37°C in LB medium including 50 μg/mL ampicillin are separated using 0.65% agarose gel including 1 or 2.5 μg/mL chroloquine.

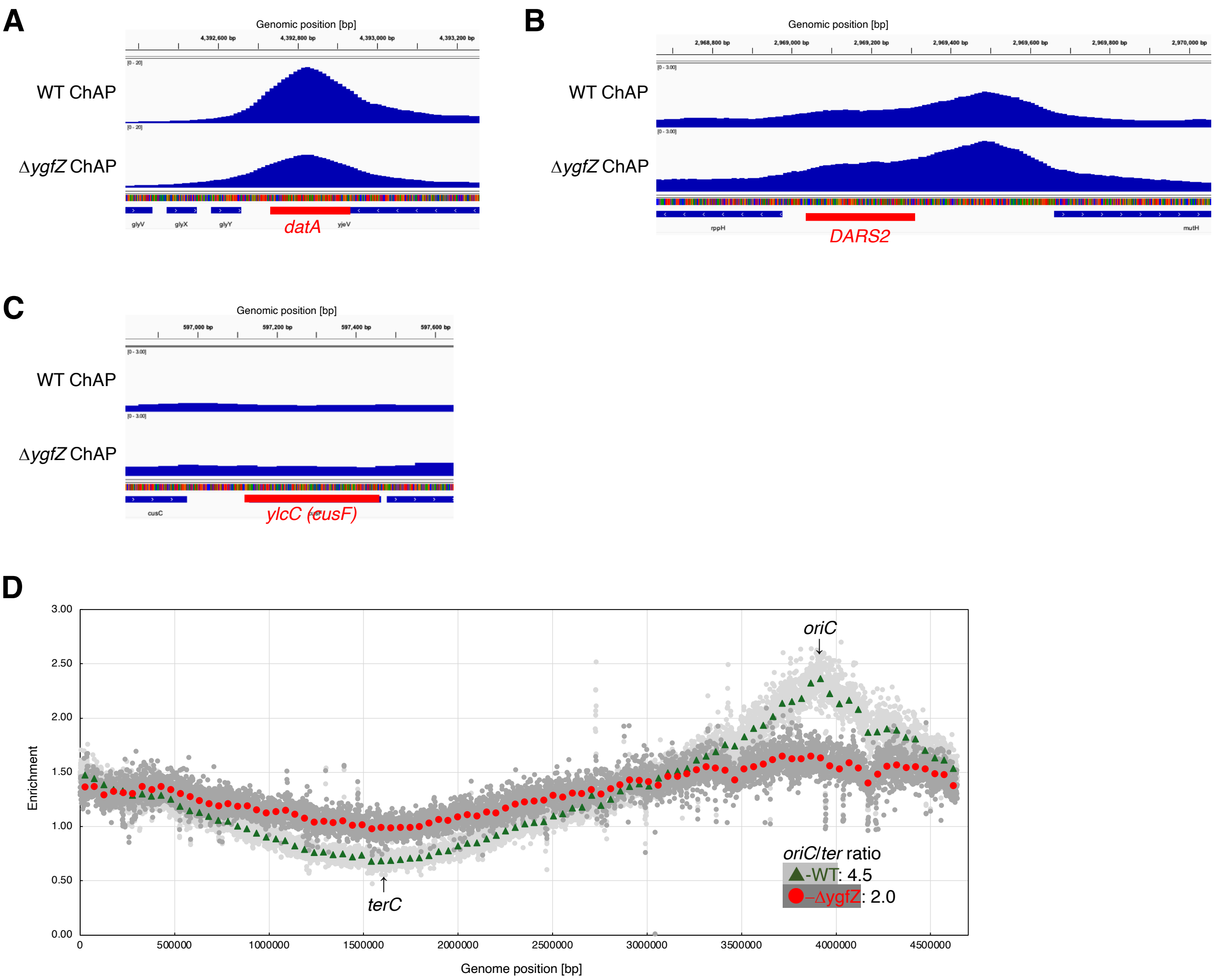

SUPPLEMENTARY FIGURE S4. Marker frequency analysis of WT and Δ*ygfZ* Input samples. (A-B) Extension of IHF binding peak observed at (A) *datA*, (B) *DARS2*, and (C) *ylcC* loci. The panels indicate IHF-binding peak (10-bp window) observed in the WT and Δ*ygfZ* ChAP dataset. At the bottom of each Figures, the location of the *datA* and *DARS2* are indicated. (D) Marker frequency analysis in WT and Δ*ygfZ* datasets. Panels indicate the enrichment of mapped reads in Input samples obtained from the same WT and Δ*ygfZ* samples as used in Fig. 5A-C. Green triangles and red circles indicate WT and Δ*ygfZ* datasets, respectively. The calculated *oriC*/*ter* ratio of WT and Δ*ygfZ* datasets are 4.5 and 2.0, respectively.

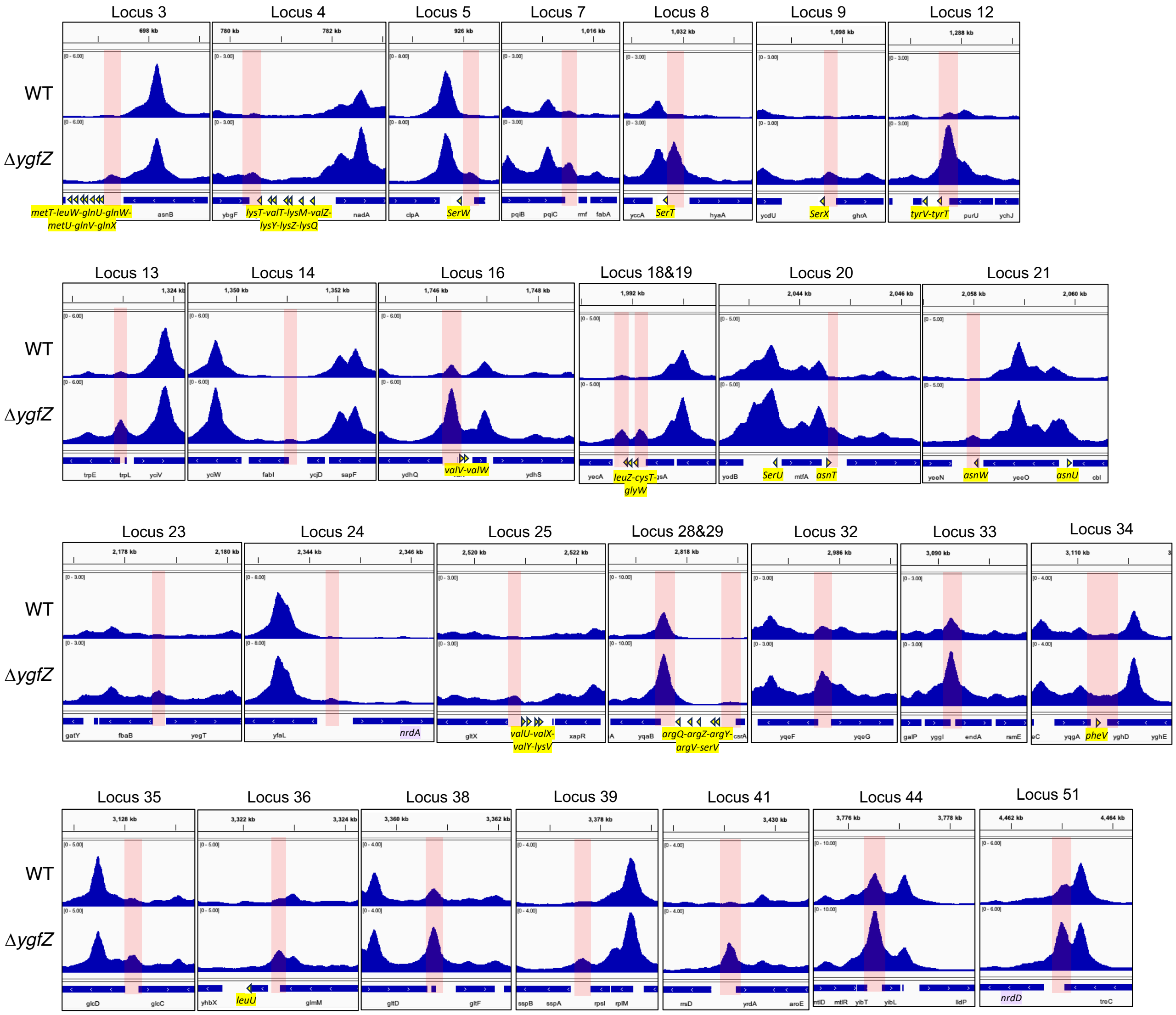

SUPPLEMENTARY FIGURE S5. The 27 chromosomal loci with the highest increase of IHF binding. The IHF binding profiles of the 27 chromosomal loci, where the highest increase of IHF ChAP/input was observed compared with WT and Δ*ygfZ* datasets, are highlighted.

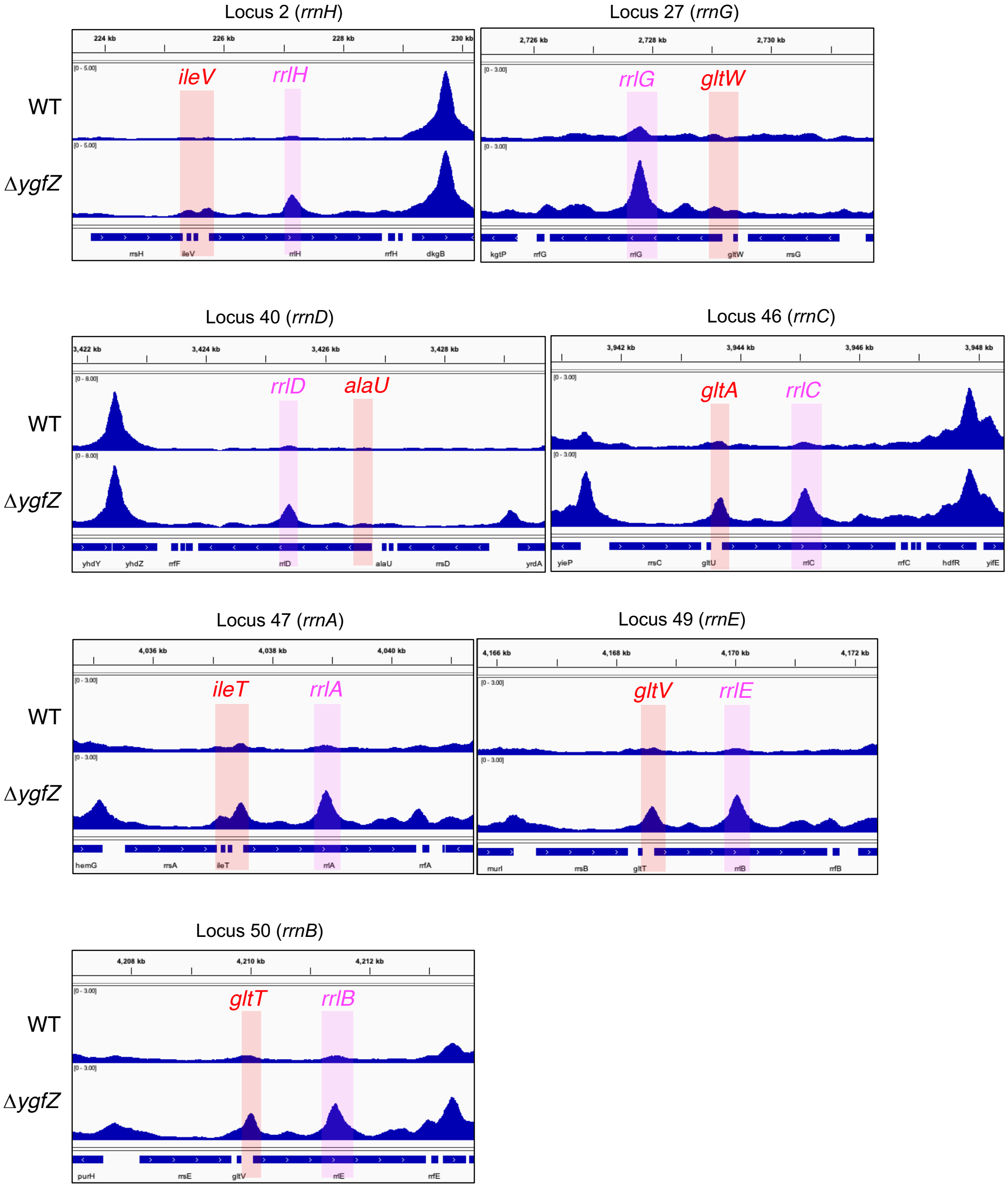

SUPPLEMENTARY FIGURE S6. The 7 ribosomal RNA operons with the highest increase of IHF binding. The IHF binding profiles of the 7 chromosomal ribosomal RNA operons, where the highest increase of IHF ChAP/input was observed compared with WT and Δ*ygfZ* datasets, are highlighted.
